# Sorghum Promotes Cell Proliferation Through Activation of the Growth Hormone/IGF-1–JAK2/STAT5b Signaling Axis *In Vitro*

**DOI:** 10.3390/biology15080594

**Published:** 2026-04-09

**Authors:** Sanghyeon Park, Dong Young Kang, Hyo Tae Kim, Woo-Shik Shin, Sangwon Lee, Jaehoon Cho, Kyoung-Jin Jang

**Affiliations:** 1 Department of Immunology, School of Medicine, Konkuk University, Chungju 27478, Republic of Korea; 2 Department of Integrative Biological Sciences and Industry, College of Life Science, Sejong University, Seoul 05006, Republic of Korea; 3 R&D Center, NEW&NEW Co., Ltd., Seoul 05855, Republic of Korea; 4 Low-Carbon Transition R&D Department, Korea Institute of Industrial Technology (KITECH), Cheonan 31056, Republic of Korea

**Keywords:** sorghum extract, IGF-1, Jak2/STAT5b, C2C12 cell, C3H10T1/2 cell

## Abstract

Sorghum is known for its anti-cancer, anti-inflammatory, and antioxidant properties, but its effect on cell growth is not well understood. First, the cytotoxicity of various sorghum extract (SE) concentrations was evaluated in C2C12 (murine myoblasts) and C3H10T1/2 (murine embryonic fibroblasts). The extent of DNA damage was then assessed, and the activation of the JAK2/STAT5b and IGF-1 pathways was observed. Studies on the transcriptional regulatory function of STAT5b revealed that SE increased STAT5b/DNA binding and transcriptional promoter activity. Consequently, STAT5b upregulation led to the increased expression of IGF-1. Moreover, other factors, such as growth hormone receptor and bone morphogenetic protein 7, were also upregulated. The results of these experiments suggest that sorghum may enhance muscle recovery or promote growth factors by stimulating the JAK2/STAT5b and IGF-1 pathways. Therefore, sorghum is expected to be an effective functional food for bone growth and muscle recovery, without inducing adverse side effects.

## Simple Summary

Sorghum is known for its anti-cancer, anti-inflammatory, and antioxidant activities, but its role in muscle cell growth remains unclear. In this study, we examined whether sorghum extract promotes the proliferation of muscle-related cells and investigated the underlying mechanisms. Sorghum extract enhanced cell growth in C2C12 and C3H10T1/2 cells without causing cytotoxic effects. Mechanistically, sorghum extract activated the JAK2/STAT5b signaling pathway and increased IGF-1 expression through enhanced STAT5b transcriptional activity. In addition, growth hormone receptor and BMP7 expression levels were also upregulated. These findings suggest that sorghum extract may support muscle regeneration and growth-related signaling pathways, indicating its potential as a safe functional food ingredient for improving muscle recovery.

## 1. Introduction

The Janus kinase/signal transducer and activator of transcription (JAK/STAT) pathway is involved in the regulation of diverse physiological and pathological processes, including cellular growth and differentiation. Among STAT family members, STAT5B has been identified as a key transcriptional regulator in growth hormone (GH)-responsive tissues, particularly in the context of skeletal and metabolic homeostasis [1,2]. Rather than acting through isolated signaling events, GH-dependent regulation of cellular growth relies on coordinated activation of downstream transcriptional programs, in which STAT5B-mediated control of insulin-like growth factor-1 (IGF-1) expression plays a central role [3].

GH is secreted predominantly by the anterior pituitary gland, and its release is influenced by multiple physiological factors, including stress, physical activity, and circadian rhythms [4,5]. In peripheral tissues, GH exerts both direct and indirect biological effects, the latter being largely mediated through IGF-1 signaling [6]. Previous studies have suggested that activation of the GH–IGF-1 axis contributes to bone and muscle development, highlighting the importance of regulatory mechanisms that modulate STAT5B-dependent transcriptional activity [7]. In this context, several natural sulfur-containing compounds have been reported to enhance GH signaling by influencing the JAK2/STAT5B pathway, indicating that bioactive substances may serve as modulators of GH-associated signaling networks [8,9].

Sorghum is a widely consumed cereal crop that is cultivated extensively in arid and semi-arid regions due to its low water requirements [10,11]. It represents an important dietary component in African and Asian populations and serves as a significant source of plant-derived nutrients [12,13,14,15]. Previous studies have demonstrated that sorghum-derived phytochemicals exhibit antioxidant, anti-inflammatory, and metabolic regulatory activities [16,17,18,19,20,21,22,23,24,25]. However, despite growing interest in the health-promoting properties of sorghum, its potential influence on hormone-related growth signaling pathways, particularly those involving GH and IGF-1, remains poorly understood.

Recent studies Sorghum contains a variety of bioactive phenolic compounds, including 3-deoxyanthocyanidins and ferulic acid, which have been reported to exhibit antioxidant, anti-inflammatory, and metabolic regulatory activities [26,27,28,29,30]. Among these compounds, 3-deoxyanthocyanidins are characteristic flavonoids in sorghum that have attracted considerable attention due to their diverse biological functions [26,27,31]. Natural polyphenols are known to modulate intracellular signaling pathways involved in cell growth and metabolism, suggesting a potential link between sorghum-derived compounds and growth-related signaling pathways. In addition, ethanol–water mixtures are widely used as extraction solvents due to their efficiency in extracting phenolic compounds with diverse polarity, supporting the use of 50% ethanol for sorghum extraction [32]. Therefore, in this study, we investigated the effects of sorghum extract on GH/IGF-1–JAK2/STAT5b signaling pathways in C2C12 and C3H10T1/2 cells.

## 2. Materials and Methods

### 2.1. Antibodies and Reagents

Cell culture reagents, including DMEM, penicillin, trypsin–EDTA (0.25%, 0.05%), and fetal bovine serum, were purchased from Gibco (Grand Island, NY, USA). Antibodies against JAK2 (#3230), pJAK2 (#3776), pSTAT5 (#9351), and pIGF-1Rβ (#3012s) were obtained from Cell Signaling Technology (Danvers, MA, USA). Antibodies recognizing β-actin (sc-47778), IGF-1Rβ (sc-713), GHR (sc-137185), and STAT5b (sc-1656), together with HRP-conjugated secondary antibodies, were sourced from Santa Cruz Biotechnology (Dallas, TX, USA). Antibodies against BMP7 (ab129156) and IGF-1 (ab9572) were acquired Abcam. Sorghum (*Sorghum bicolor* L., cv. ‘Nampungchal’, a glutinous cultivar developed and registered by the Rural Development Administration (RDA, Republic of Korea; application No. 2013-246) and cultivated in Hamyang, Republic of Korea) grains used for sorghum extract (SE) preparation were procured from a local market (Hamyang, NongHyup, Republic of Korea) in the Republic of Korea. The extract was prepared by incubating sorghum in 50% (*v*/*v*) ethanol at room temperature for 72 h. The extract was filtered, concentrated (at 60 °C for 2 h), and freeze-dried (at −80 °C for 24 h). The concentrations of sorghum extract (SE) used in this study were defined based on the dry weight of the crude extract after solvent evaporation. The 50% ethanol extract of sorghum is known to contain various phenolic compounds, including 3-deoxyanthocyanidins (e.g., luteolinidin and apigeninidin), phenolic acids, and flavonoids, which are associated with its antioxidant and anti-inflammatory activities.

### 2.2. Cell Viability Assay

C2C12 and C3H10T1/2 cells were seeded into 96-well plates and incubated for stabilization. Cells were subsequently treated with increasing concentrations of SE (10–400 μg/mL) for 24 h. Cell viability was evaluated using an MTT-based assay, and absorbance was measured at 560 nm. Results were expressed as mean ± SEM from independent experiments.

### 2.3. Cell Culture

C2C12 myoblasts (CRL-1772; ATCC, Manassas, VA, USA) and C3H10T1/2 murine embryonic fibroblasts (KCLB-10226; Korean Cell Line Bank, Seoul, Republic of Korea) were maintained in media (DMEM supplemented with 10% FBS, 1% penicillin (37 °C, 5% CO_2_)). C2C12 cells at passage 8 and C3H10T1/2 cells at passage 11 were used for all experiments. For the experiment, cells at 70–80% confluence were washed with phosphate-buffered saline prior to treatment. For differentiation studies, cells at 70% confluence were transferred to media and treated with SE.

### 2.4. Chromatin Immunoprecipitation (ChIP) Assay

ChIP analysis was carried out using a commercial kit (Sigma-Aldrich; Merck KGaA, Darmstadt, Germany) according to the protocol. Cells were seeded of 2 × 10^6^ cells in 100 mm culture dishes, differentiated for 48 h, and subsequently exposed to sorghum extract (SE; 80 μg/mL) for an additional 24 h prior to cross-linking. Protein–DNA complexes were fixed using formaldehyde and quenched with glycine, followed by cell lysis and chromatin shearing via sonication (25% amplitude with 30 s on/off cycles for 20 min on ice). Following centrifugation, the clarified chromatin fraction was diluted at a 1:1 ratio with dilution buffer and incubated with anti-STAT5b antibodies in Parafilm-coated wells for 90 min. Immunoprecipitation controls included normal goat IgG and anti-RNA polymerase II antibodies. After extensive washing, immunocomplexes were treated with proteinase K to reverse cross-links, and the recovered DNA was purified using GenElute Binding Columns. Enriched DNA fragments were quantified by quantitative PCR (qPCR) using IGF-1-specific primers (sense, 5′-CCACACACACCTATTCACCC-3′ and antisense, 5′-CCTGGAGCCATAGGGTATGA-3′). qPCR was performed in a 20 μL SYBR Green PCR Master Mix under the following protocol.

### 2.5. Comet Assay

Cells were treated with SE and subjected to comet assay analysis according to the manufacturer’s instructions (ab238544; Abcam, Cambridge, UK). DNA damage was visualized using fluorescence microscopy.

### 2.6. DAPI Staining

Cells were exposed to SE and stained with DAPI to assess nuclear morphology. Fluorescence images were obtained using a fluorescence microscope (Olympus, Tokyo, Japan).

### 2.7. Quantitative Real-Time qPCR Analysis

Total RNA was isolated and reverse-transcribed into cDNA. Gene (IGF-1) expression was analyzed by qPCR and normalized to GAPDH. Total RNA was extracted from C2C12 and C3H10T1/2 cells using the RNeasy Mini Kit (#74103; Qiagen GmbH, Hilden, Germany), and RNA concentration was determined spectrophotometriccally (at 230 nm). RNA concentration and purity were determined using a spectrophotometer (NanoDrop 1000; Thermo Fisher Scientific, Waltham, MA, USA), and the A260/A280 ratio ranged between 1.82 and 2.0, indicating acceptable RNA quality. cDNA was synthesized via reverse transcription using oligo(dT) primers. Following incubation at 42 °C for 1 h and enzyme inactivation at 95 °C for 5 min. Quantitative PCR (qPCR) was performed to evaluate IGF-1 expression and gene expression levels were normalized to GAPDH. Amplification was carried out on a LightCycler 480 system (Roche, Basel, Switzerland) under the following cycling conditions: 40 cycles of denaturation at 95 °C for 30 s, annealing at 58 °C for 30 s, and extension at 72 °C for 30 s. IGF-1 transcript levels were normalized to glyceraldehyde-3-phosphate dehydrogenase (GAPDH) expression. The primer sequences used were as follows: IGF-1 sense 5′-TCGGTGCCTCAGTTTTCCTC-3′ and antisense 5′-GATGTTGCACCCTCCTGGAA-3′; GAPDH sense 5′-TTCACCACCATGGAGAAGGC-3′ and antisense 5′-AGTGATGGCATGGACTGTGG-3′.

### 2.8. Total Cell Lysis and Western Blotting

Cellular proteins were extracted by incubating the samples in RIPA buffer (Gibco-BRL; Thermo Fisher Scientific, Grand Island, NY, USA), containing a cocktail of protease and phosphatase inhibitors. The total protein content of each lysate was quantified via the Bradford method. Equal amounts of protein (30 µg) were mixed with loading buffer and denatured at 100 °C for 5 min. Proteins were separated by electrophoresis on 10% SDS–polyacrylamide gels at a constant voltage (70–100 V) for 120 min and transferred onto PVDF membranes using a wet transfer system at 175 V for 60 min. Membranes were incubated with specific primary and secondary antibodies, and signals were detected using a chemiluminescence system. Protein levels were quantified using ImageJ software (version 1.53, National Institutes of Health, Bethesda, MD, USA).

### 2.9. Statistical Analysis

All data are presented as mean ± SEM. Differences between groups were evaluated using either Student’s *t*-test or one-way analysis of variance (ANOVA), as appropriate. When multiple group comparisons were required, statistical significance was determined using one-way ANOVA followed by Tukey’s post hoc test. Statistical significance was considered at *p* < 0.05, which is a widely accepted criterion for biological experiments. Data analyses were carried out using SAS software (version 9.3).

## 3. Results

### 3.1. SE Induces C2C12 and C3H10T1/2 Cell Proliferation in a Concentration-Dependent Manner

**Figure 1:**
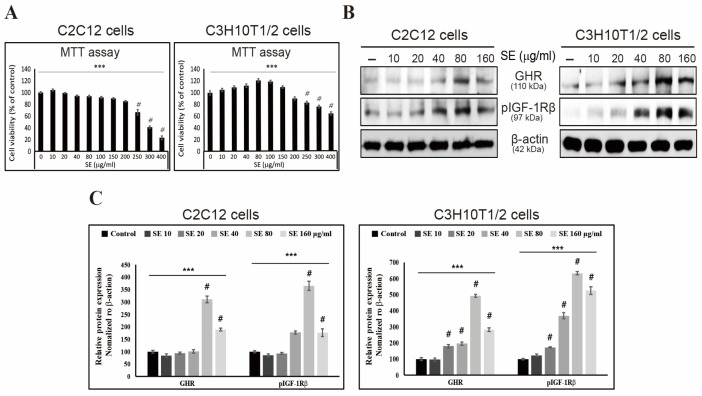
Effects of SE on cell viability and protein expression in C2C12 and C3H10T1/2 cells. (**A**) Cell viability was measured by MTT assay after 24 h of SE treatment (0–400 μg/mL). (**B**) Protein expression levels of GHR and pIGF-1Rβ were analyzed by Western blot. (**C**) Quantification of protein expression normalized to β-actin. Data are presented as mean ± SEM (*n* = 3). *** *p* < 0.001 (ANOVA test), # *p* < 0.001 vs. control. The original uncropped Western blot images corresponding to Figure 1B are provided in the Supplementary Materials.

SE was extracted in 50% ethanol and allowed to react for 72 h because of its poor solubility. After filtration, only the liquid was used. To evaluate the effect of SE on cells, MTT assays were performed on C2C12 and C3H10T1/2 cells at SE concentrations ranging from 0 to 400 μg/mL. We found that there was no significant cytotoxicity up to 200 μg/mL, whereas at higher concentrations, cell death was induced (Figure 1A). Based on the MTT assay results, we treated the cells with SE to analyze the expression of GHR and pIGF-1Rβ using Western blot analysis. The results showed that the expression levels increased with higher SE concentrations and then decreased at 160 μg/mL (Figure 1B,C).

### 3.2. Ethanol-Extracted SE Modulates Proliferation-Related Protein Expression in C3H10T1/2 Cells

**Figure 2:**
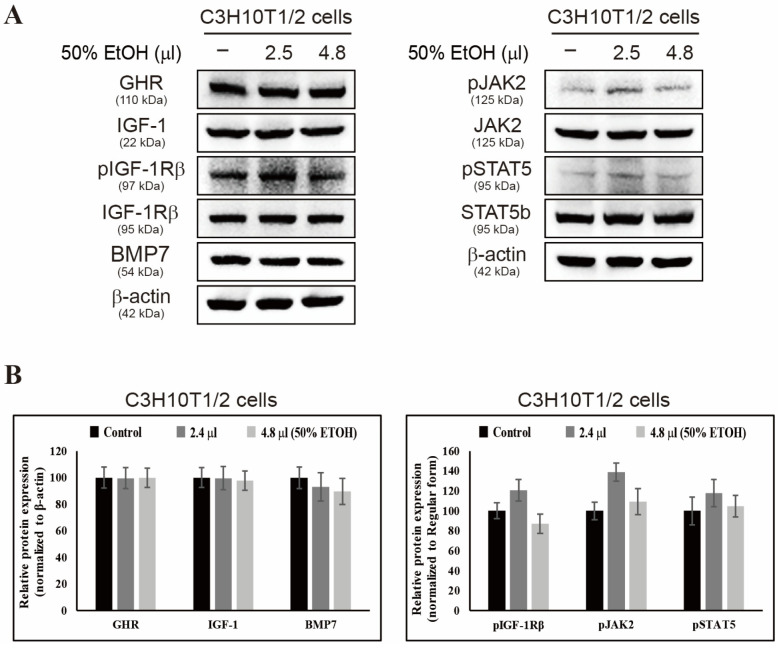
Effect of ethanol vehicle on protein expression in C3H10T1/2 cells. Cells were treated with equivalent volumes of 50% ethanol. (**A**) Protein expression was analyzed by Western blot. (**B**) Densitometric analysis of the Western blot results from (**A**) was performed and normalized to β-actin and the corresponding regular form. Data are presented as mean ± SEM (*n* = 3). The original uncropped Western blot images corresponding to Figure 2A are provided in the Supplementary Materials.

The highest expression of GHR, IGF-1, and pSTAT5b in C3H10T1/2 cells was observed at 80 μg/mL of SE, which was therefore selected as the most effective concentration for signaling in subsequent experiments. A concentration of 250 μg/mL SE was included as a negative control for comparative purposes. To exclude potential effects of the solvent, cells were also treated with volumes of 50% ethanol corresponding to those used for SE 40 and 80 μg/mL. These treatments resulted in no significant changes in protein expression compared with the blank control (Figure 2A,B), indicating that the observed effects were attributable to SE itself rather than the solvent. This careful control confirms that the modulation of proliferation-related protein expression reflects the biological activity of the extract.

### 3.3. SE Is Not Cytotoxic at the Appropriate Concentration

**Figure 3:**
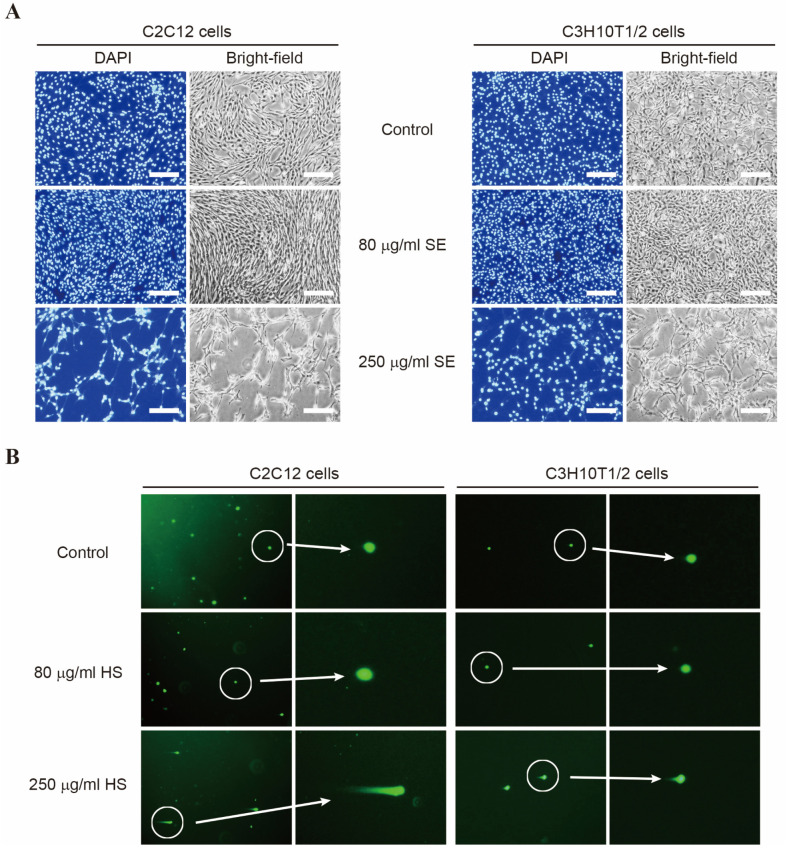
Assessment of cytotoxicity and DNA damage following SE treatment. (**A**) Nuclear morphology was evaluated by DAPI staining. Scale bar = 200 μm. (**B**) DNA damage was assessed using a comet assay. The data represents the results of representative images.

For further cytotoxicity experiments, we evaluated cell death in C2C12 and C3H10T1/2 cells treated with 80 μg/mL and 250 μg/mL of SE, using morphological analysis with DAPI staining (Figure 3A). We observed an increase in the cell number at a concentration of 80 μg/mL, whereas at 250 μg/mL of SE, a decrease in the cell number was observed. Because high concentrations of SE treatment may lead to DNA damage, we performed a comet assay to determine whether SE induces DNA damage. Fluorescence microscopy revealed an increase in the comet lengths and the number of comet-positive cells at 250 μg/mL of SE, whereas no comets were formed at 80 μg/mL (Figure 3B). These results indicate that SE at moderate concentrations is not cytotoxic and promotes cell proliferation.

### 3.4. SE Increases the Expression of GHR, IGF-1, and the JAK2/STAT5b Pathway

**Figure 4:**
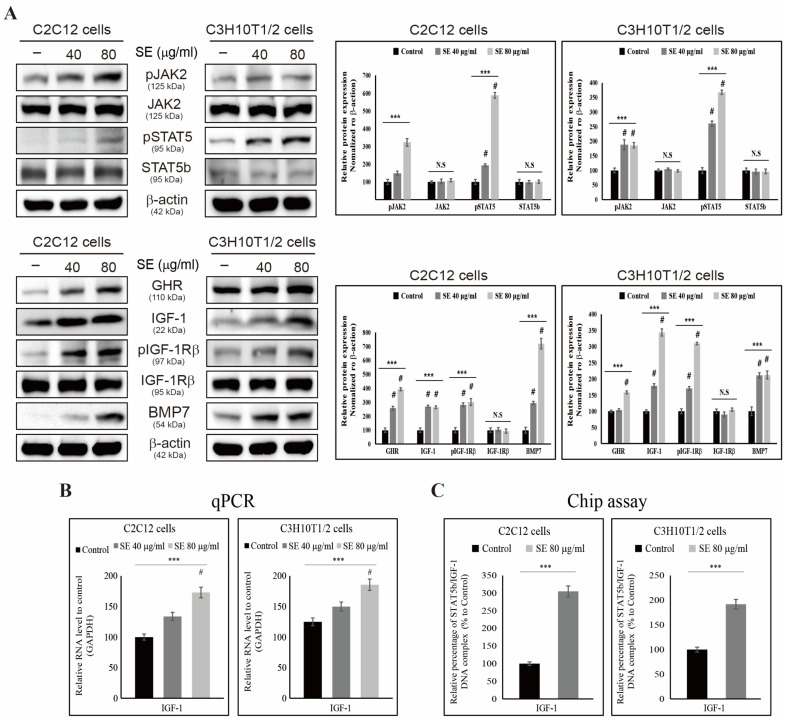
Activation of GH/IGF-1–JAK2/STAT5b signaling by SE. (**A**) Protein expression levels were analyzed by Western blot. *** *p* < 0.001 (ANOVA test), # *p* < 0.001 vs. control. N.S., not significant. (**B**) The expression levels of IGF-1 mRNA were detected by real-time qPCR after sorghum extract treatment at the indicated concentrations for 24 h. Data are representative of three independent experiments. *** *p* < 0.001 (ANOVA test), # *p* < 0.001 vs. control. (**C**) STAT5b binding to the IGF-1 promoter was assessed by ChIP assay. Data are representative of three independent experiments. *** *p* < 0.001 versus control (*t*-test). The original uncropped Western blot images corresponding to Figure 4A are provided in the Supplementary Materials.

**Figure 5:**
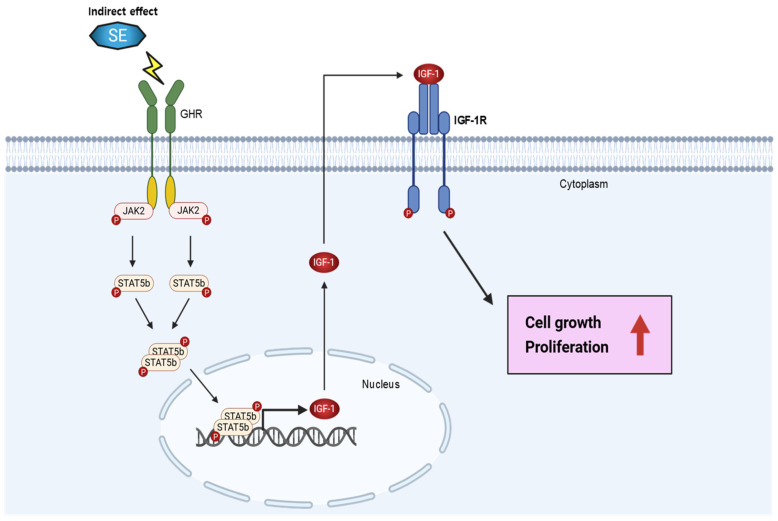
Proposed mechanism of sorghum extract (SE)-mediated regulation of muscle cell proliferation via the JAK2/STAT5b–IGF-1 signaling pathway. SE treatment activates the JAK2/STAT5b signaling cascade, leading to phosphorylation and dimerization of STAT5b, followed by its translocation into the nucleus. Activated STAT5b promotes the transcription of IGF-1, resulting in increased IGF-1 production. Secreted IGF-1 subsequently binds to its receptor (IGF-1R), triggering downstream signaling pathways that contribute to enhanced cell growth and proliferation. Black arrows indicate the direction of signaling flow, whereas the red arrow indicates increased cellular response associated with cell growth and proliferation.

Western blotting was used to analyze the translational expression of GHR, IGF-1, and JAK2/STAT5b in the C2C12 and C3H10T1/2 cells treated with 40 and 80 μg/mL of SE. The results revealed that SE increased the expression of GHR, pIGF-1Rβ, bone morphogenetic protein 7 (BMP7), pJAK2, pSTAT5, and IGF-1 without inducing changes in the total JAK2, STAT5b, or IGF-1Rβ (Figure 4A). This indicates that SE stimulates cell signaling by activating key growth hormones. To further confirm the ability of SE to induce GH signaling, we examined the expression of IGF-1 mRNA, which was found to also be increased by SE (Figure 4B). We next examined the effect of SE on STAT5b binding to the IGF-1 promoter region. ChIP analysis with a STAT5b-specific antibody demonstrated that treatment with 80 μg/mL SE enhanced STAT5b binding to the IGF-1 promoter, leading to the formation of a STAT5b–IGF-1 complex and subsequent transcriptional activation (Figure 4C). These results imply that pSTAT5 and IGF-1 serve as primary molecular targets of SE, and that SE treatment promotes their expression by upregulating IGF-1 transcription. Collectively, these findings suggest that SE may act as an activator of critical growth hormone (GH) signaling pathways (Figure 5).

## 4. Discussion

Sorghum is a commonly consumed crop that is easily accessible, making it the focus of various studies. Recently, research has confirmed the antimicrobial effects of sorghum phenolic extracts as well as their anti-diabetic properties [33,34]. In addition, the effects of another variety of sorghum, *Hwanggeumchal sorghum* extract, on bone growth have been investigated [35]; however, this study demonstrates for the first time that sorghum ethanol extract regulates growth-related signaling pathways in C2C12 and C3H10T1/2 cells, highlighting a previously unknown mechanism.

Metabolic diseases are increasingly recognized as complex disorders involving interconnected molecular and signaling networks rather than single pathological pathways [36,37]. Systems biology and bioinformatics-based studies have revealed extensive crosstalk between metabolic dysregulation, inflammation, and hormone-related signaling, highlighting the necessity of multi-target therapeutic approaches [38,39]. Recently, a systems biology and bioinformatics-based study analyzing the molecular interplay between COVID-19 and diabetic nephropathy identified shared regulatory networks and hub genes that connect inflammatory, metabolic, and immune pathways, which further demonstrates that complex diseases arise from integrated network disturbances rather than isolated mechanisms [40]. In this context, natural products such as sorghum, which contain diverse bioactive compounds, may represent promising candidates for modulating multifaceted metabolic disease networks.

In particular, bone growth research has garnered considerable interest in East Asia, where individuals tend to have smaller body frames, thus leading to various studies in this area. The aim has been to find sources from natural compound foods, and sorghum presents such an advantage. As a result, while testing various natural substances, a hypothesis was formed that SE could indirectly promote growth. It was hypothesized that IGF-1 could function as a growth factor and induce GH signaling. Generally, GH is important for cell growth and differentiation [41], and GH binds to GHR, thereby promoting cell growth [42]. Similarly, studies have revealed that methylsulfonylmethane (MSM) can enhance GH signaling by regulating it through the Jak2/STAT5b pathway [9]. In addition, an increase in GH signaling has been demonstrated by NTS, as it was shown to enhance GH signaling in C2C12 cells [8].

IGF-1 binds to either the insulin receptor (IR) or IGF-1R to transmit signals to cells and primarily influences growth, development, and differentiation in adults [43]. Both IGF-1R and IR are expressed in almost all tissues; however, variability depends on the specific cells where the receptors are present [44]. IGF-1 overexpression promotes cell growth through the regulation of Jak2 and STAT5b, which consequently induces the expression of GH, thereby further promoting cell growth [45]. STAT5b acts as an IGF-1 transcription factor and, upon phosphorylation, forms dimers to induce its function [46]. In this study, SE was found to regulate the expression of pSTAT5 and IGF-1 to upregulate the levels of GHR, pSTAT5, pJak2, and IGF-1. Growth hormone (GH) exerts its effects by binding to the growth hormone receptor (GHR), which leads to receptor dimerization and activation of the associated Janus kinase 2 (JAK2). Activated JAK2 subsequently phosphorylates and activates STAT5b, which dimerizes and translocates to the nucleus to act as a transcription factor. One of the major transcriptional targets of STAT5b is IGF-1, which mediates the downstream effects of GH by promoting cell proliferation and differentiation [3]. These findings suggest that SE may function in a similar manner to that of GH. Furthermore, it was observed that SE increased the formation of the STAT5b protein and IGF-1 DNA complexes, thus confirming that enhanced STAT5b signaling may be mediated by IGF-1.

STAT5b plays a critical role in cell growth, and IGF-1 strongly depends on STAT5b phosphorylation [47]. In the cell growth studies using SE, high concentrations (250 μg/mL) led to significant cell death, as observed via DAPI staining. This suggests that an optimal concentration (80 μg/mL) of SE is required for balanced cell growth. At an optimal concentration of SE, the pSTAT5 protein was overexpressed, and notably, cells with increased pSTAT5 levels exhibited elevated levels of GHR, pJak2, and BMP7. BMPs are known to play a crucial role in muscle regeneration and bone formation, processes that are closely associated with IGF-1–mediated growth signaling [3,48,49,50]. Although the direct interaction between BMP7 and the IGF-1 pathway was not examined in the present study, the concurrent upregulation of these factors suggests a potential synergistic contribution to muscle and bone-related growth responses. These results suggest that SE enhances GH signaling primarily through the regulation of the Jak2/STAT5b/IGF-1 pathway, thereby indicating its potential to improve GH signaling.

Although the present study employed a crude ethanol extract of sorghum rather than isolated constituents, previous phytochemical analyses have identified 3-deoxyanthocyanidins and ferulic acid as major bioactive polyphenols in sorghum extracts prepared under similar extraction conditions [26,27]. In particular, ferulic acid has been shown to activate receptor-mediated signaling cascades associated with JAK/STAT and IGF-1 regulation [28,29,30], suggesting a potential mechanistic link to the enhanced JAK2/STAT5b/IGF-1 signaling observed in the present study. Therefore, while the current findings support a growth-promoting role of SE via the JAK2/STAT5b/IGF-1 axis, further studies using purified compounds will be necessary to delineate the individual contributions of these bioactive constituents.

Notably, the bell-shaped dose–response observed in this study, with maximal signaling activation at 80 μg/mL followed by attenuation at higher concentrations, is a common phenomenon in natural product research. Such non-linear responses are often attributed to the complex composition of crude extracts, where synergistic effects at moderate concentrations may be counterbalanced by antagonistic interactions or physicochemical limitations, including reduced solubility or aggregation, at higher doses [51,52].

This study demonstrated the beneficial effects of SE on cell proliferation and recovery *in vitro*, providing evidence of its potential as a source of bioactive compounds for therapeutic or cosmeceutical applications. These findings suggest that 40 μg/mL may represent the minimum effective concentration and that 80 μg/mL may represent the highest expression of key signaling proteins. While the present study focused on myoblast proliferation, muscle growth is a complex process involving both proliferation and differentiation. Key regulators such as myostatin and follistatin play critical roles in muscle development, and future studies should investigate the effects of sorghum extract on myogenic differentiation and these regulatory factors. However, the absence of a fully characterized dose–response curve limits our ability to precisely define the minimum effective concentration and the maximum non-toxic concentration of SE. Further investigations using in vivo models are required to comprehensively evaluate the pharmacokinetic properties, physiological relevance, systemic efficacy, and safety of sorghum extract. These studies will contribute to a more comprehensive understanding of the biological functions of SE and help to clarify its clinical applicability and safety profile.

## 5. Conclusions

SE can upregulate the expression of IGF-1 by modulating the Jak2/STAT5b signaling pathway in C2C12 mouse myoblasts and C3H/10T1/2 mouse embryonic fibroblasts. Specifically, pSTAT5b functions as a direct transcription factor for the IGF-1 gene, thus playing a key role in enhancing GH signaling. This regulation is critical for the amplification of IGF-1 expression, which is essential for mediating the downstream effects of GH. Finally, the 50% ethanol used for sorghum extract had no effect on cell proliferation.

## Data Availability

The data presented in this study are available on request from the corresponding author. The data are not publicly available due to personal reasons.

## Supplementary Materials

The following supporting information can be downloaded at: https://www.mdpi.com/article/10.3390/biology15080594/s1, Original images for W.B_Biology.
