# Supplementary figures and images for: Sorghum Promotes Cell Proliferation Through Activation of the Growth Hormone/IGF-1–JAK2/STAT5b Signaling Axis In Vitro
Source: Biology (Basel). 2026 Apr 9;15(8):594. doi: 10.3390/biology15080594 (PMC13112945; doi:10.3390/biology15080594)

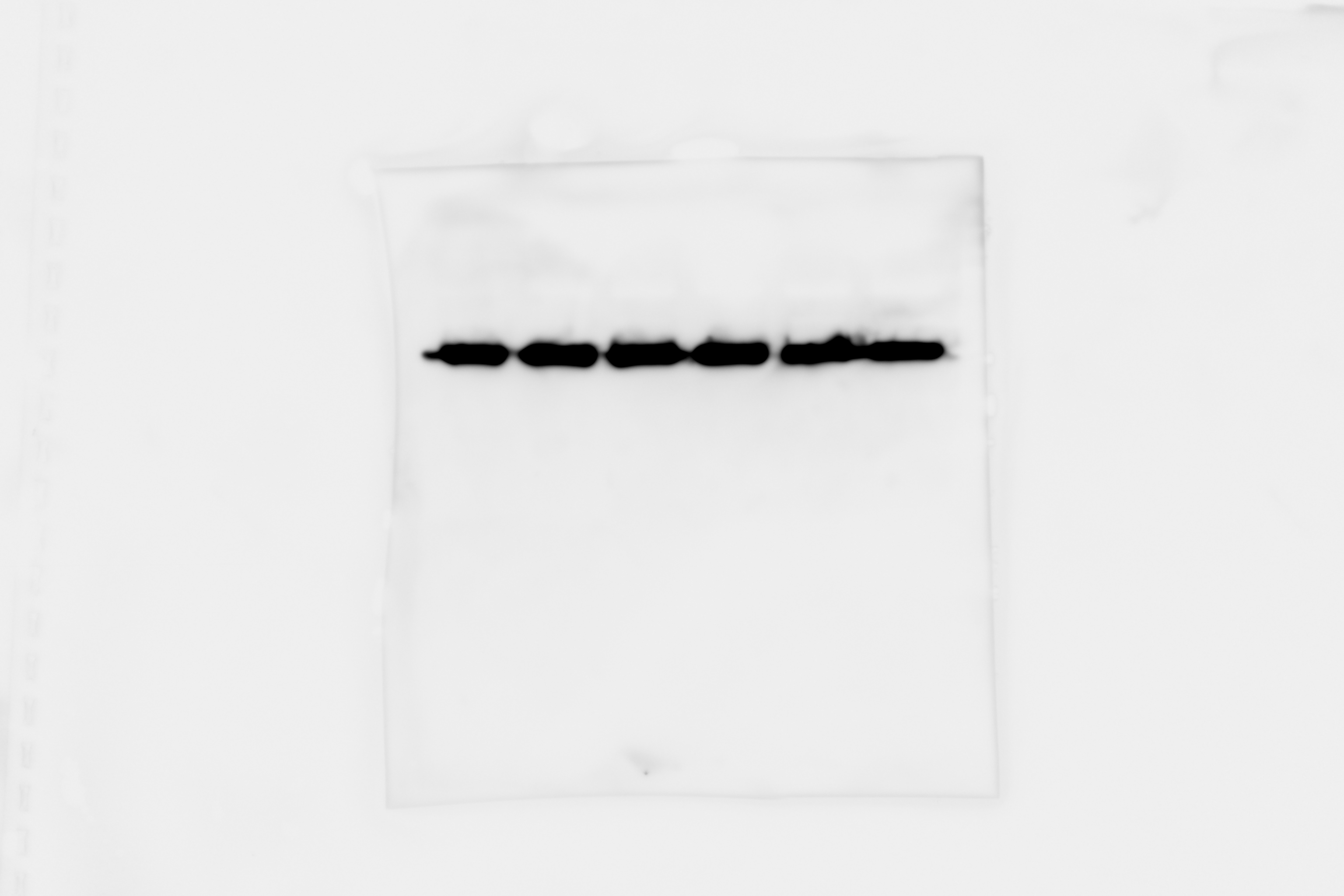

Supplement: Supplementary file 1 [file biology-15-00594-s001.zip › biology-4201171-supplementary/Original images for W.B_Biology/Fig.1B_C2C12/Fig.1B_C2C12_beta-actin_v2.tif]

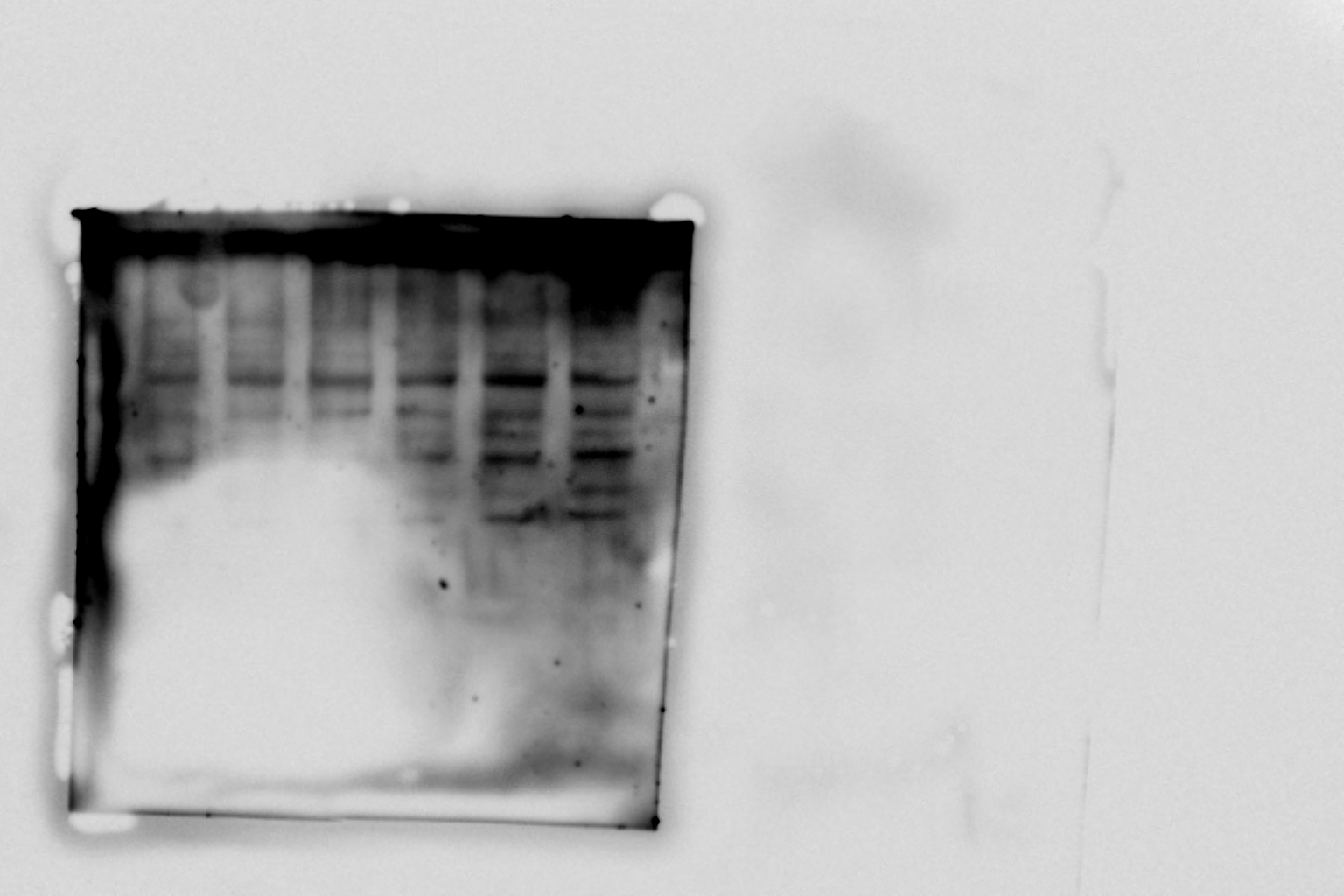

Supplement: Supplementary file 1 [file biology-15-00594-s001.zip › biology-4201171-supplementary/Original images for W.B_Biology/Fig.1B_C2C12/Fig.1B_C2C12_GHR_v2.tif]

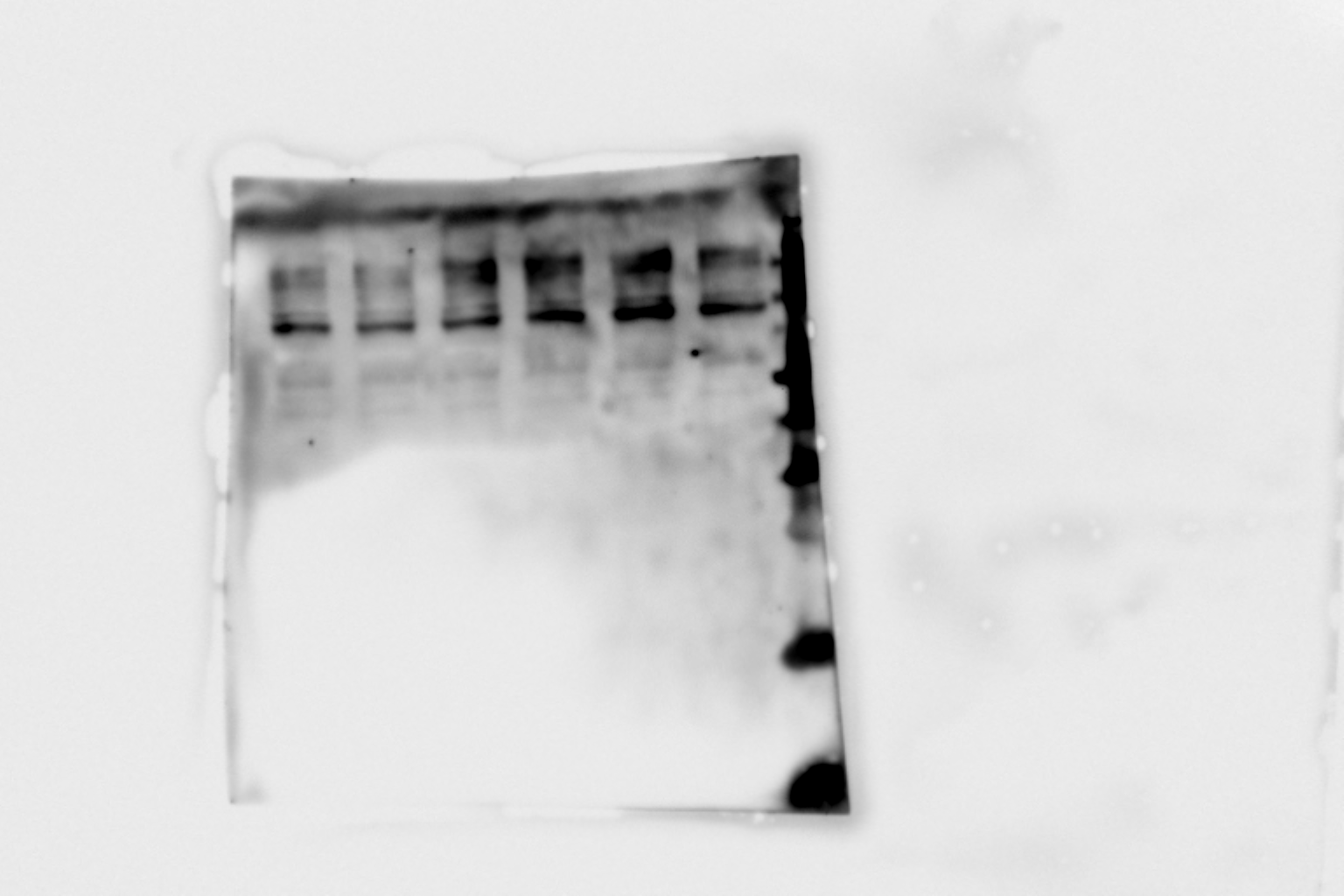

Supplement: Supplementary file 1 [file biology-15-00594-s001.zip › biology-4201171-supplementary/Original images for W.B_Biology/Fig.1B_C2C12/Fig.1B_C2C12_pIGF-1Rbeta_v2.tif]

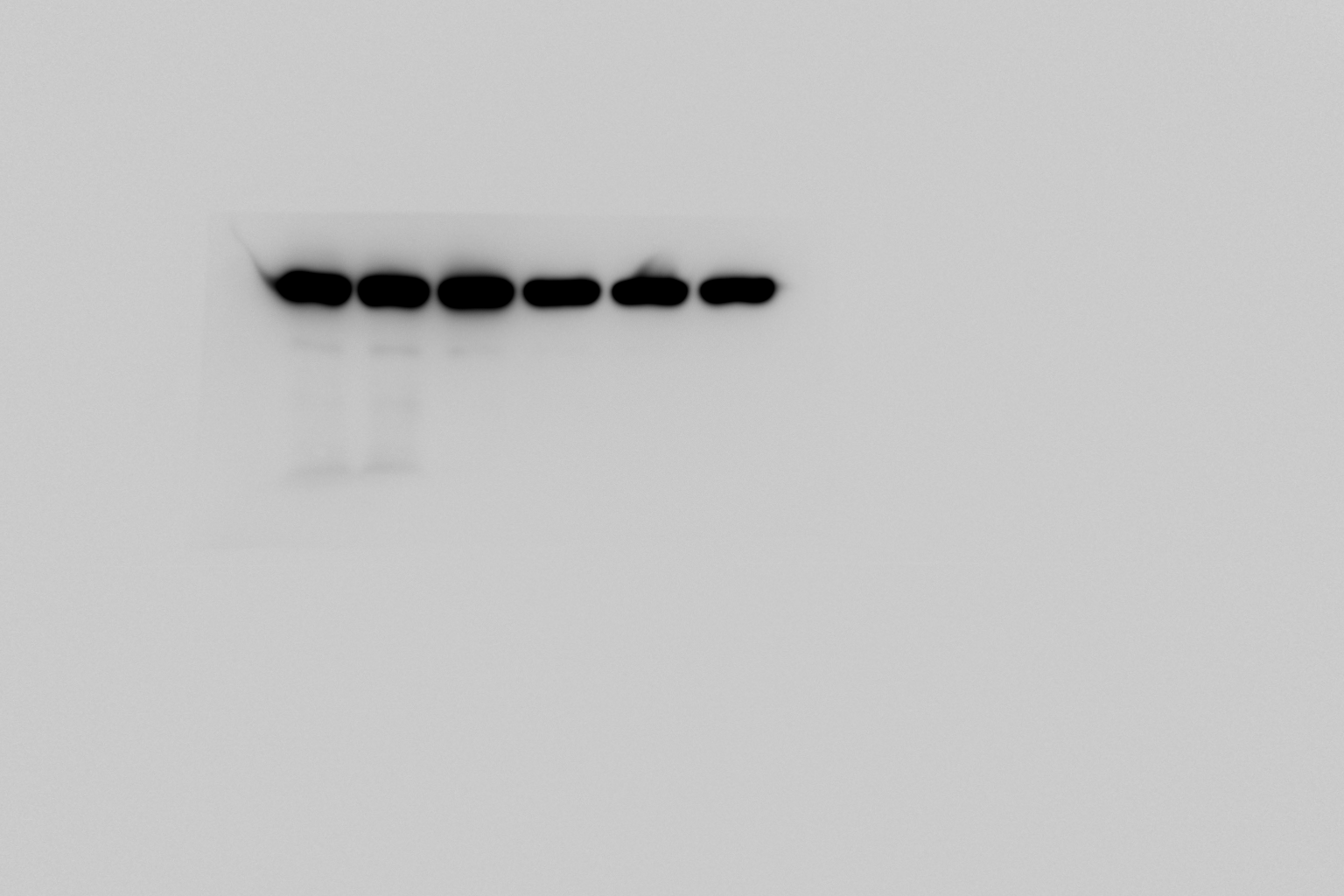

Supplement: Supplementary file 1 [file biology-15-00594-s001.zip › biology-4201171-supplementary/Original images for W.B_Biology/Fig.1B_C3H10/Fig.1B_C3H10_beta-actin_v2.tif]

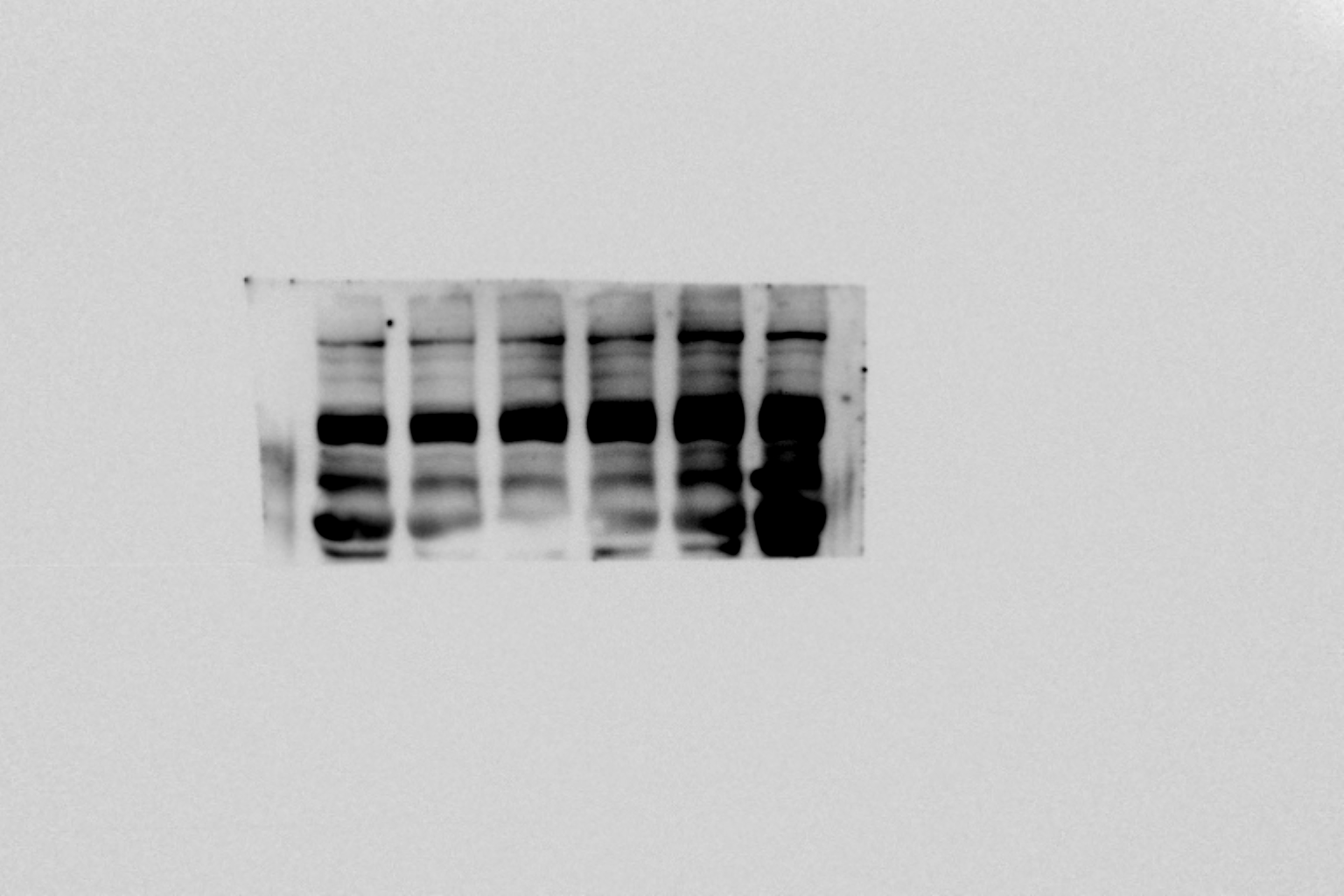

Supplement: Supplementary file 1 [file biology-15-00594-s001.zip › biology-4201171-supplementary/Original images for W.B_Biology/Fig.1B_C3H10/Fig.1B_C3H10_GHR_v2.tif]

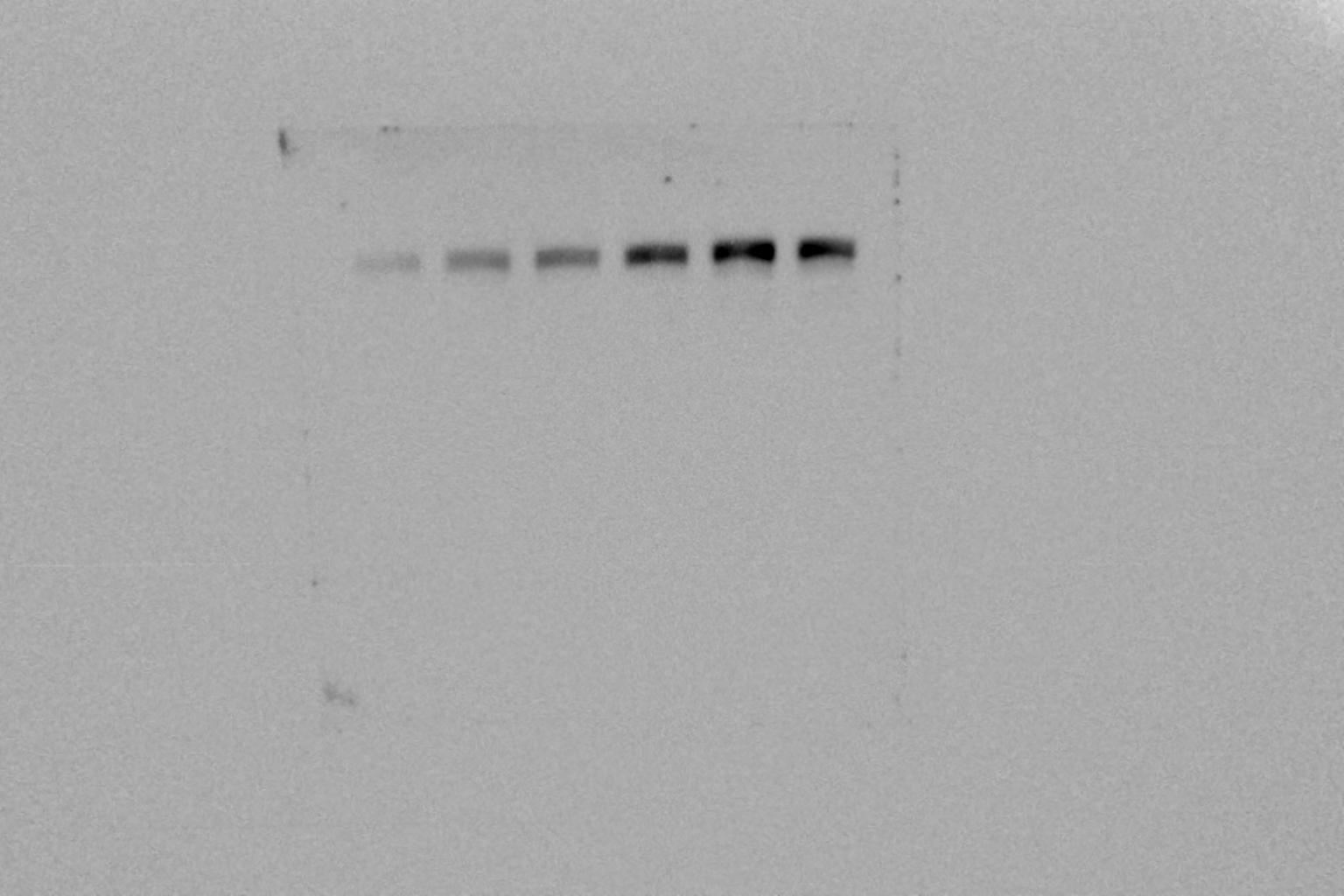

Supplement: Supplementary file 1 [file biology-15-00594-s001.zip › biology-4201171-supplementary/Original images for W.B_Biology/Fig.1B_C3H10/Fig.1B_C3H10_pIGF-1Rbeta_v2.tif]

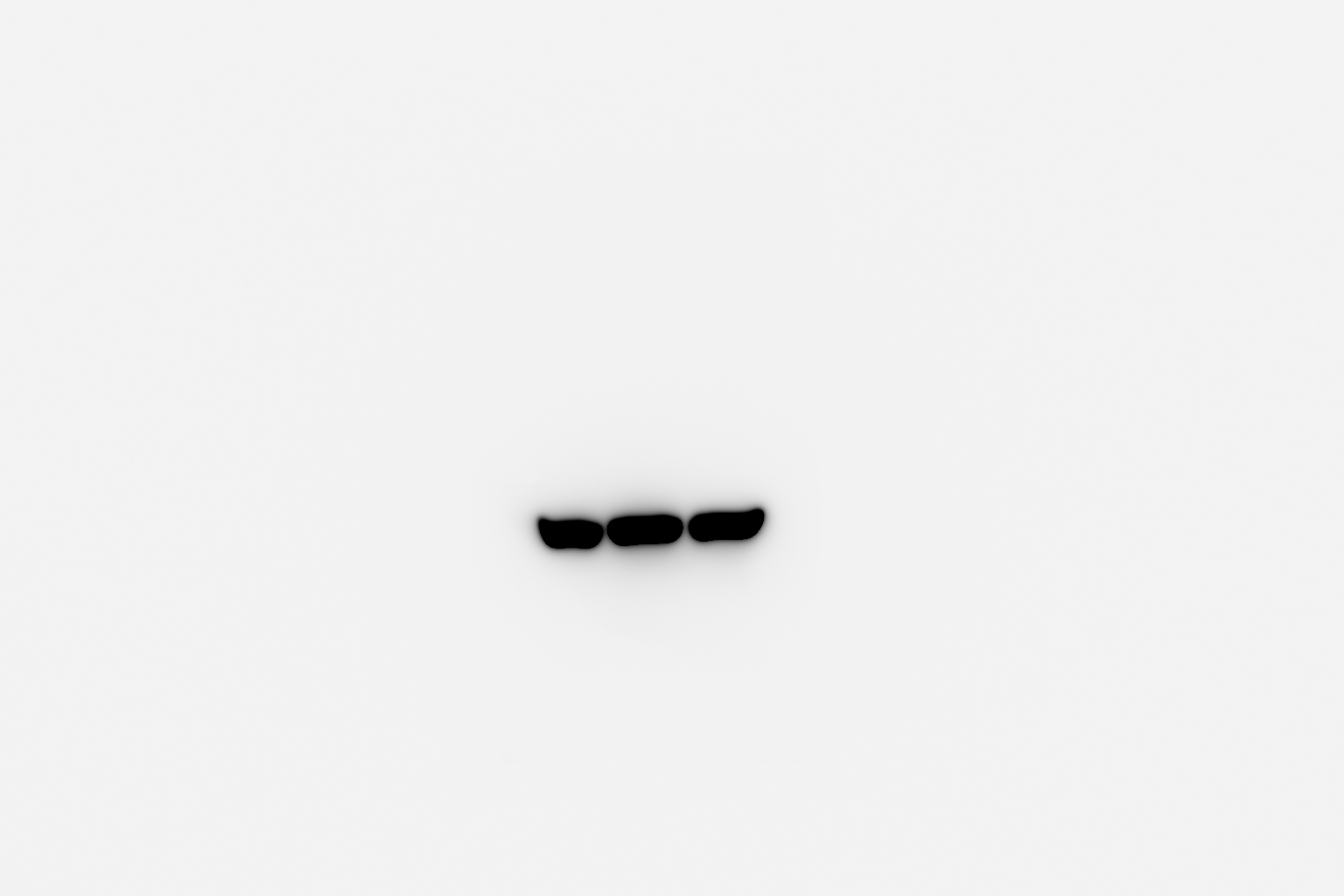

Supplement: Supplementary file 1 [file biology-15-00594-s001.zip › biology-4201171-supplementary/Original images for W.B_Biology/Fig.4A_C2C12/Fig.4A_C2C12_beta-actin-1_v2.tif]

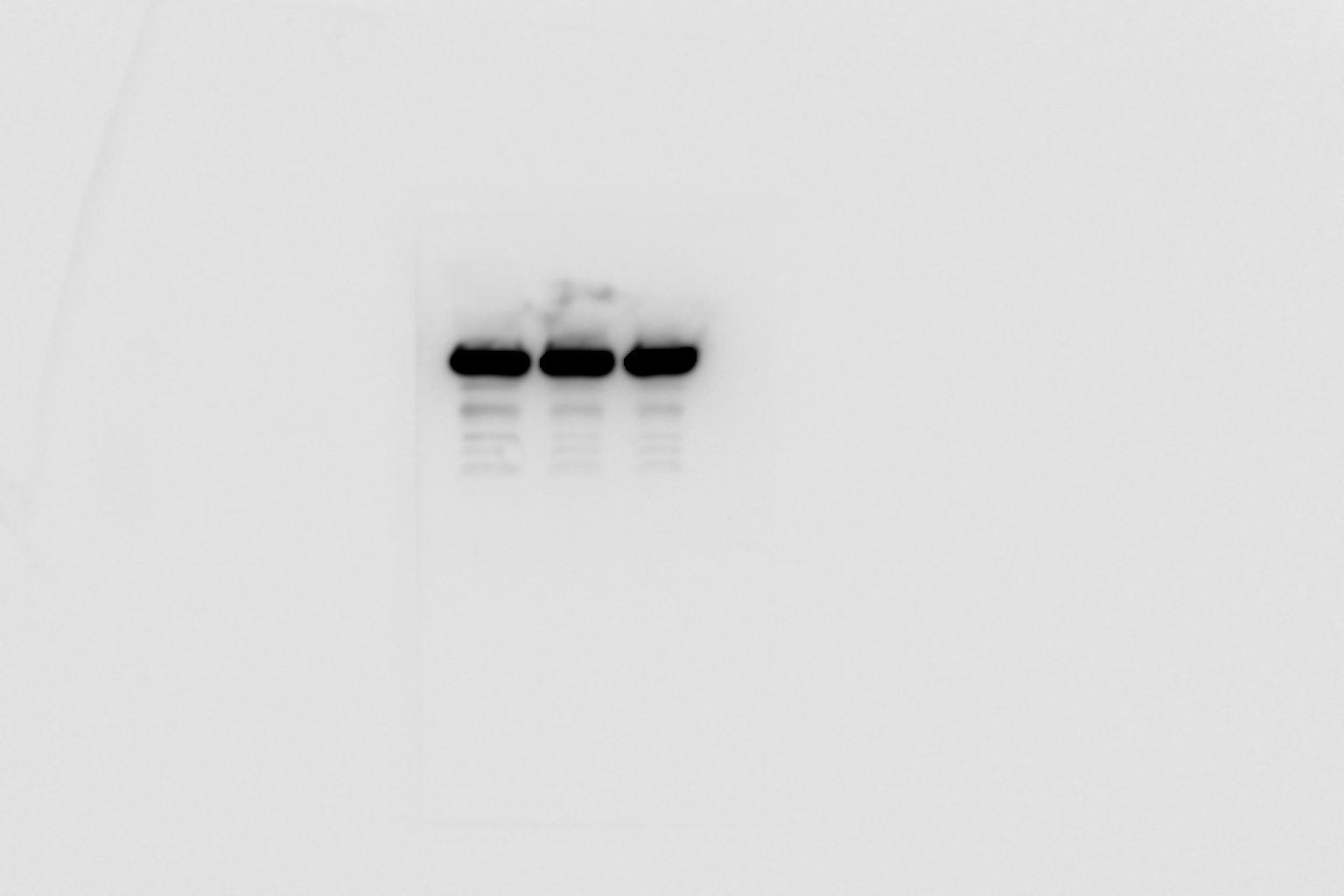

Supplement: Supplementary file 1 [file biology-15-00594-s001.zip › biology-4201171-supplementary/Original images for W.B_Biology/Fig.4A_C2C12/Fig.4A_C2C12_beta-actin-2_v2.tif]

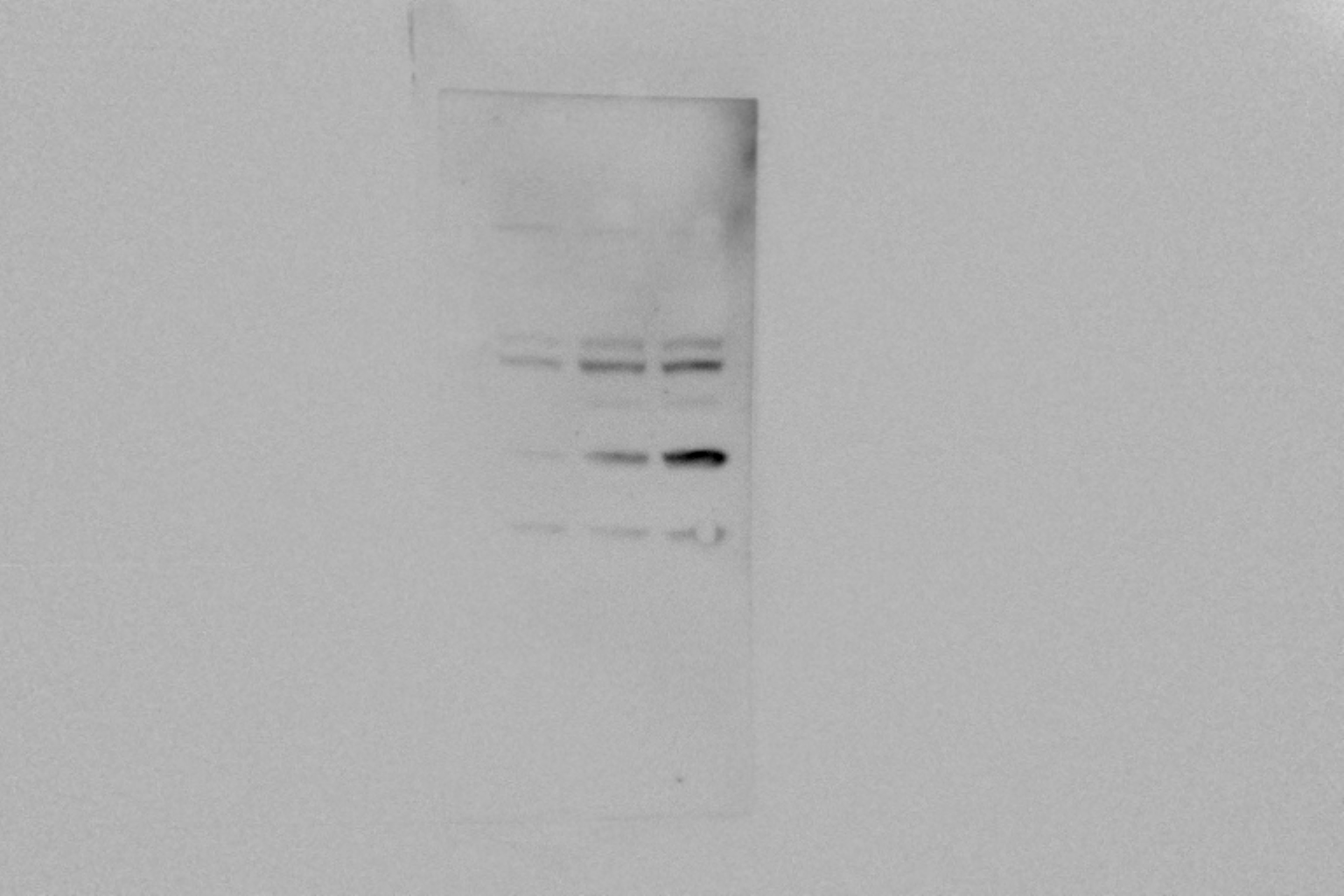

Supplement: Supplementary file 1 [file biology-15-00594-s001.zip › biology-4201171-supplementary/Original images for W.B_Biology/Fig.4A_C2C12/Fig.4A_C2C12_BMP7_v2.tif]

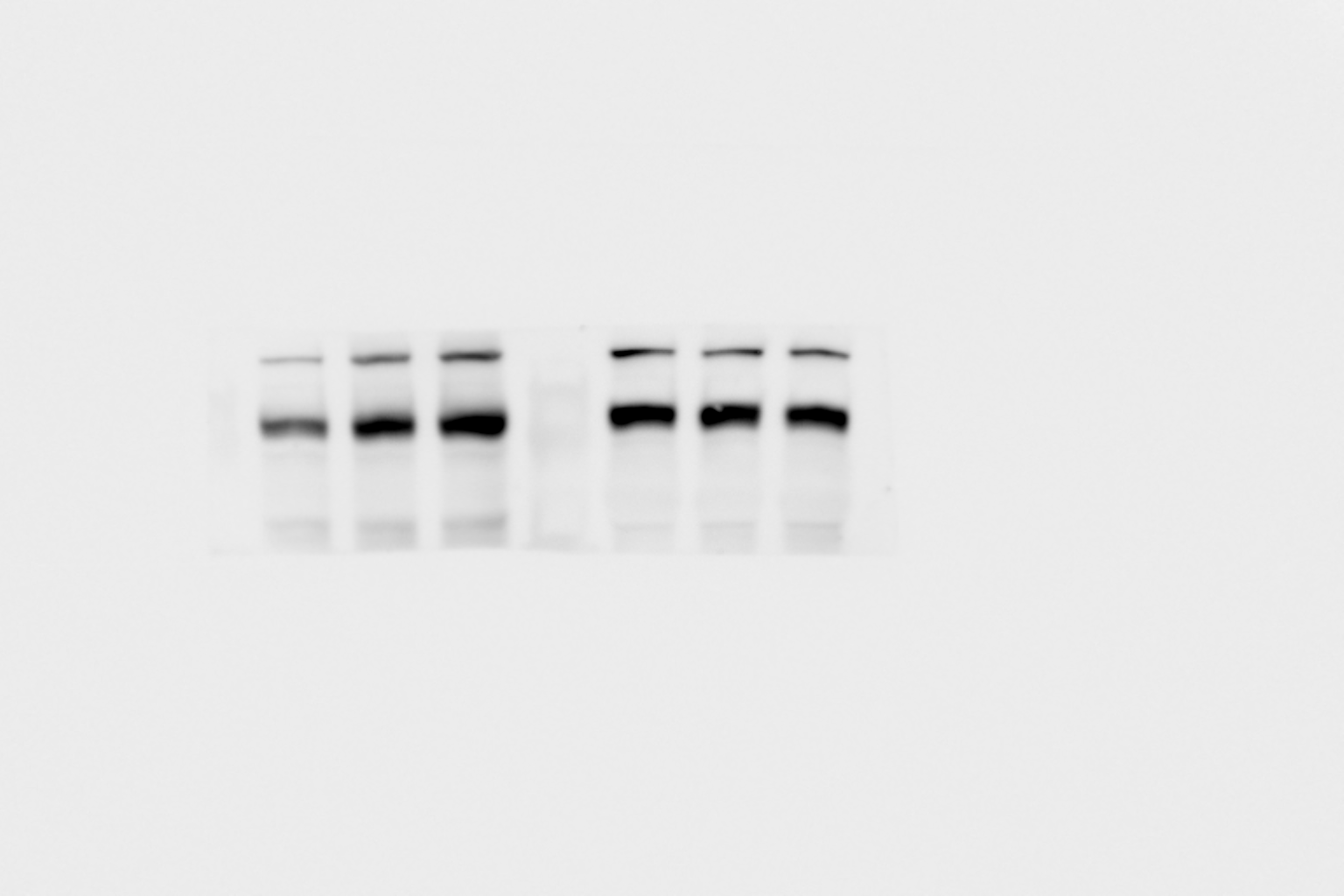

Supplement: Supplementary file 1 [file biology-15-00594-s001.zip › biology-4201171-supplementary/Original images for W.B_Biology/Fig.4A_C2C12/Fig.4A_C2C12_GHR_v2.tif]

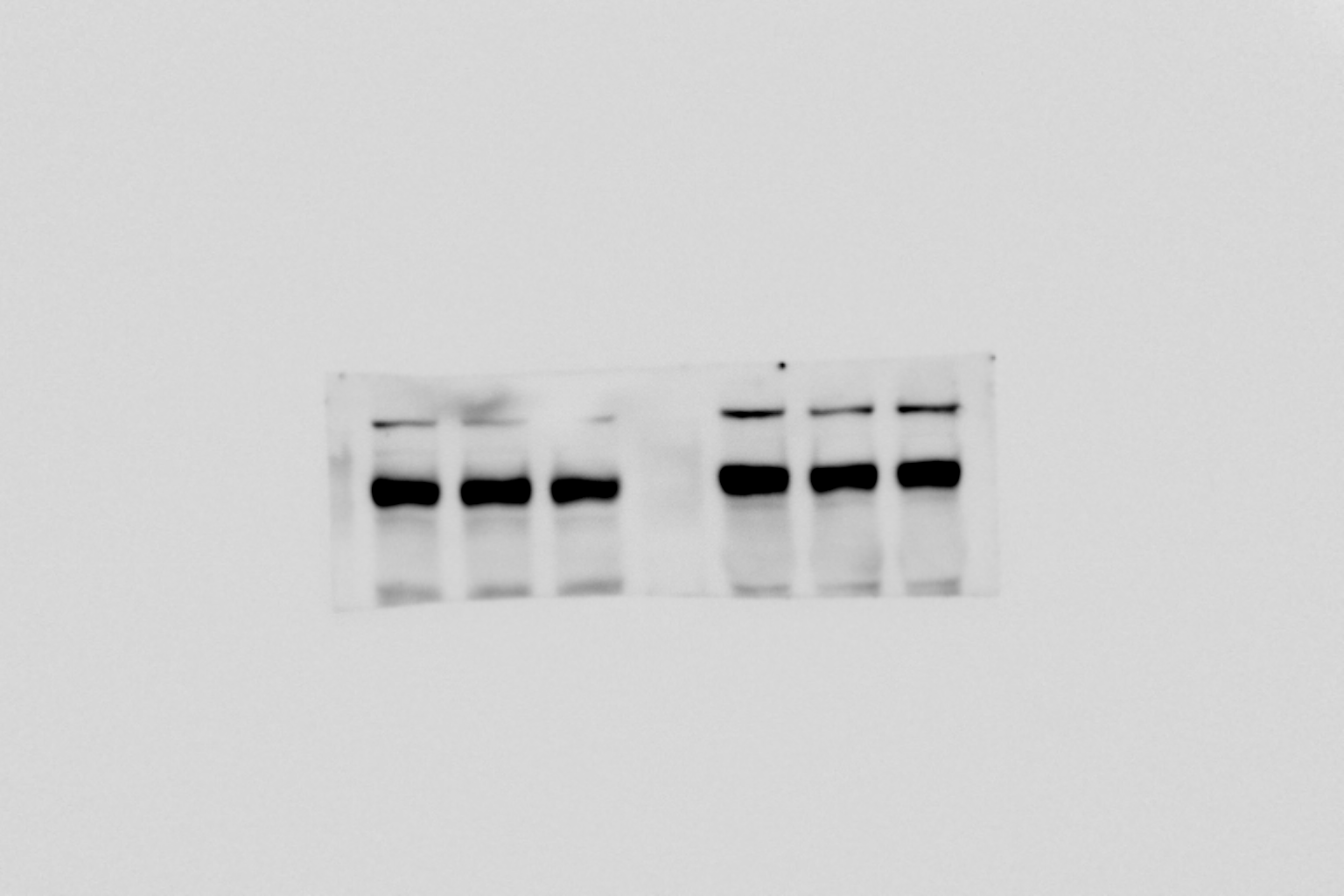

Supplement: Supplementary file 1 [file biology-15-00594-s001.zip › biology-4201171-supplementary/Original images for W.B_Biology/Fig.4A_C2C12/Fig.4A_C2C12_IGF-1Rbeta_v2.tif]

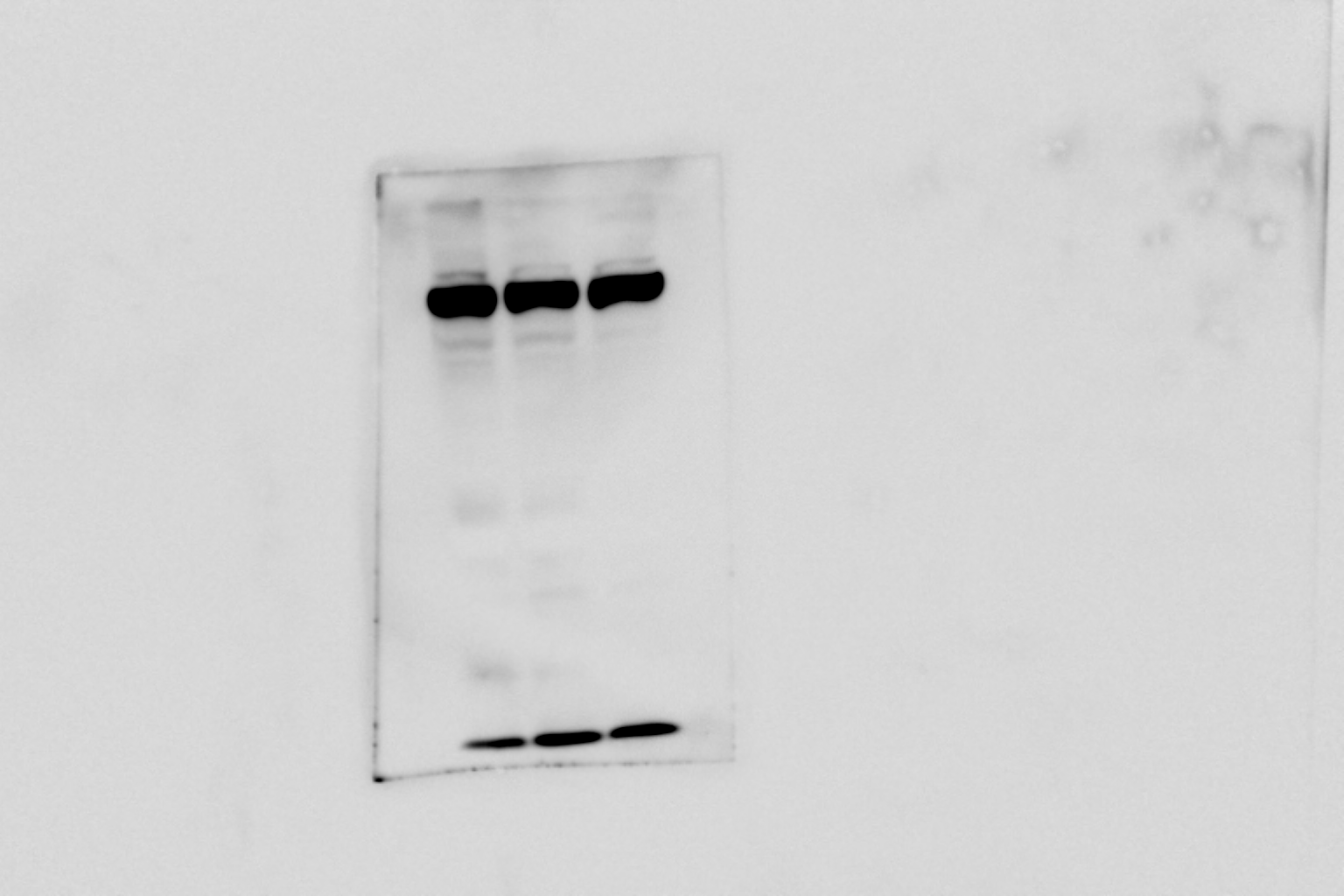

Supplement: Supplementary file 1 [file biology-15-00594-s001.zip › biology-4201171-supplementary/Original images for W.B_Biology/Fig.4A_C2C12/Fig.4A_C2C12_IGF-1_v2.tif]

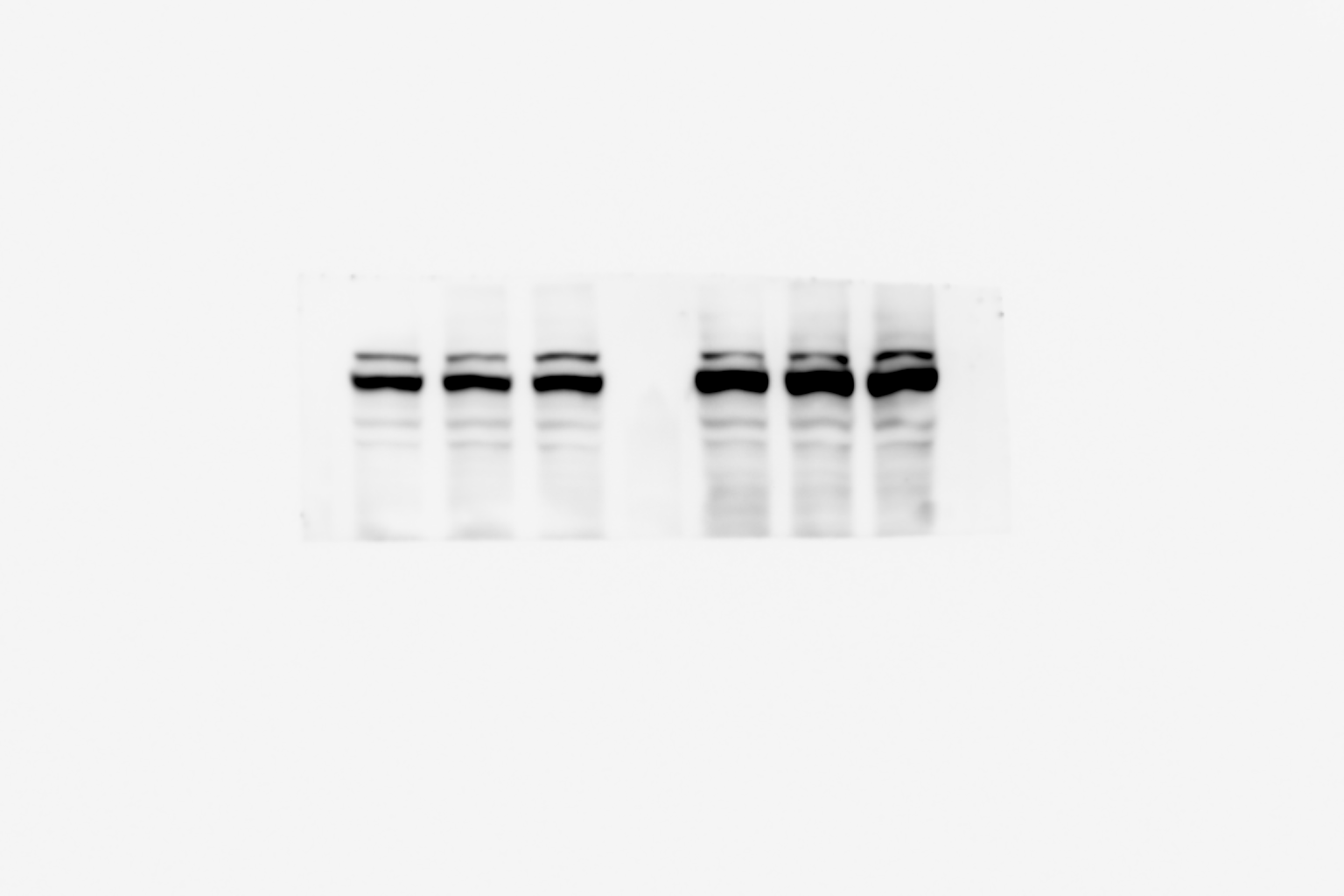

Supplement: Supplementary file 1 [file biology-15-00594-s001.zip › biology-4201171-supplementary/Original images for W.B_Biology/Fig.4A_C2C12/Fig.4A_C2C12_JAK2_v2.tif]

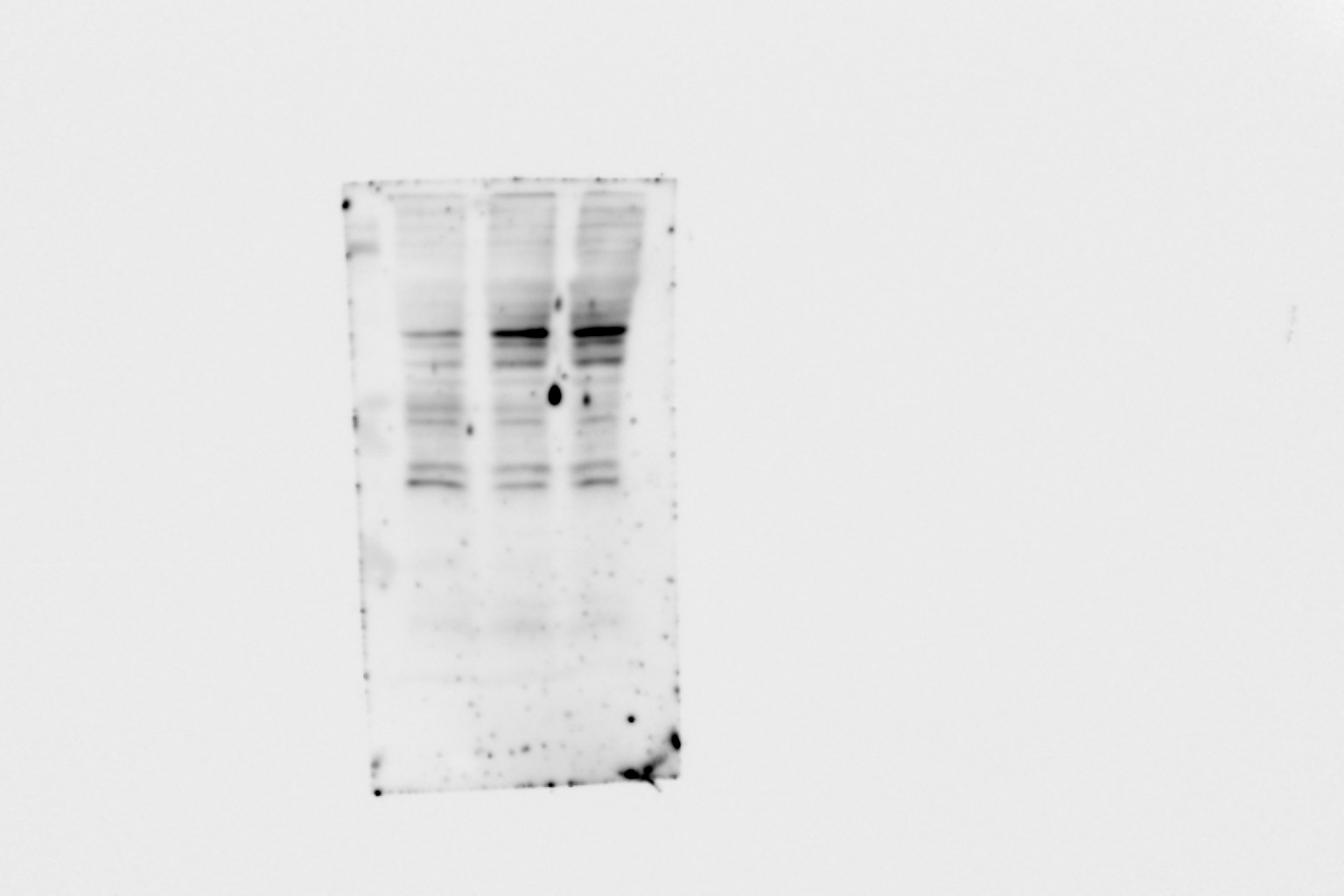

Supplement: Supplementary file 1 [file biology-15-00594-s001.zip › biology-4201171-supplementary/Original images for W.B_Biology/Fig.4A_C2C12/Fig.4A_C2C12_pIGF-1Rbeta_v2.tif]

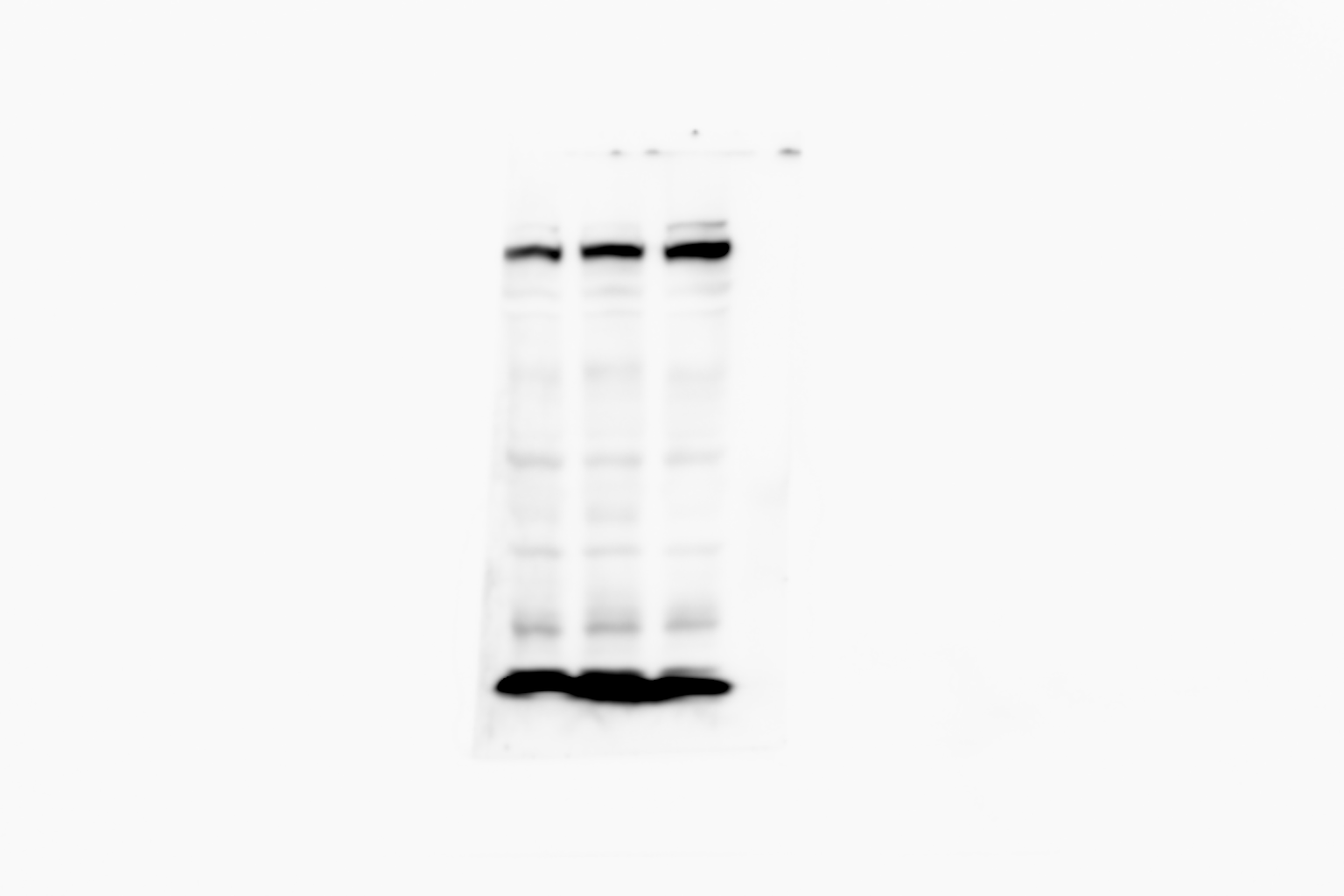

Supplement: Supplementary file 1 [file biology-15-00594-s001.zip › biology-4201171-supplementary/Original images for W.B_Biology/Fig.4A_C2C12/Fig.4A_C2C12_pJAK2_v2.tif]

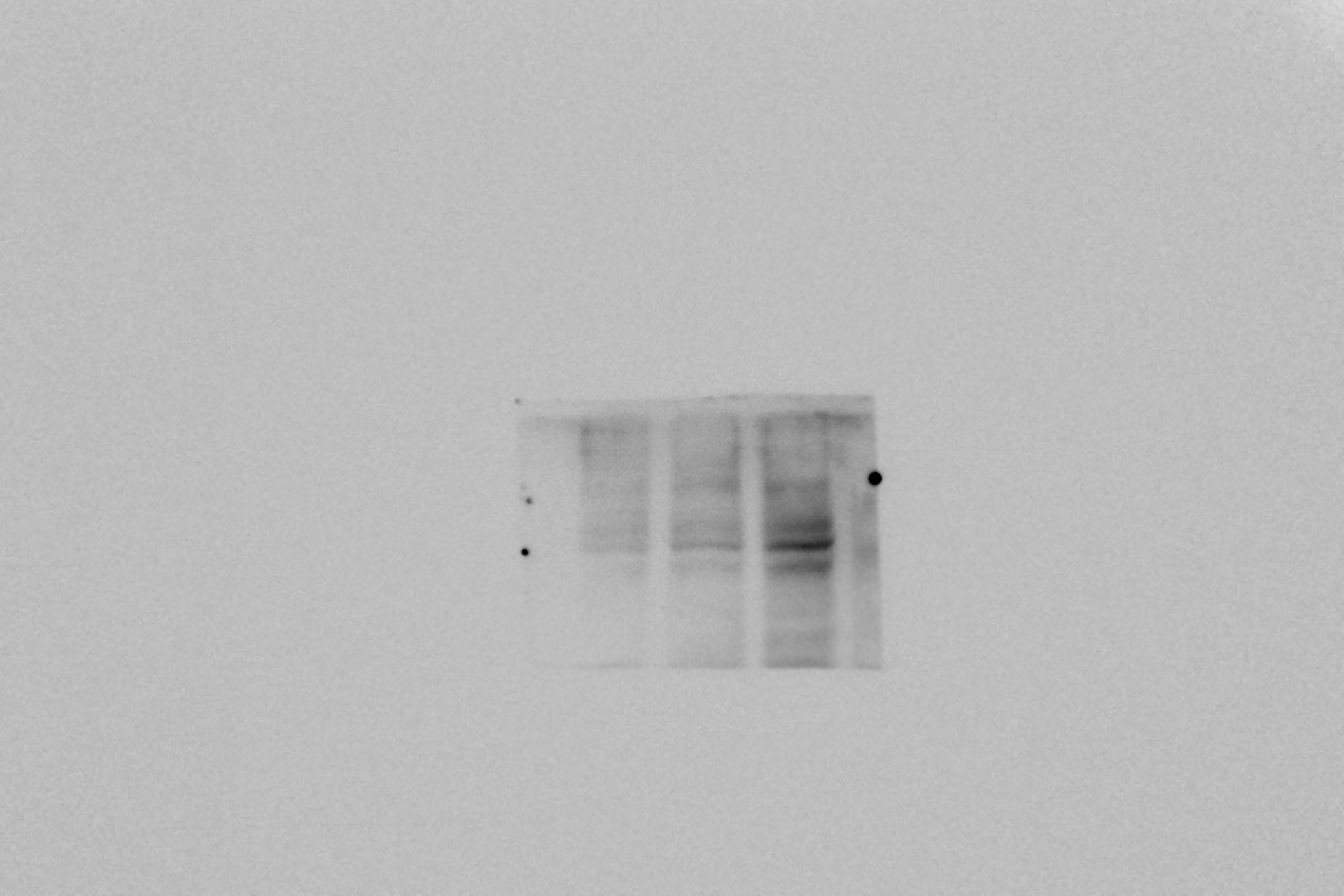

Supplement: Supplementary file 1 [file biology-15-00594-s001.zip › biology-4201171-supplementary/Original images for W.B_Biology/Fig.4A_C2C12/Fig.4A_C2C12_pSTAT5_v2.tif]

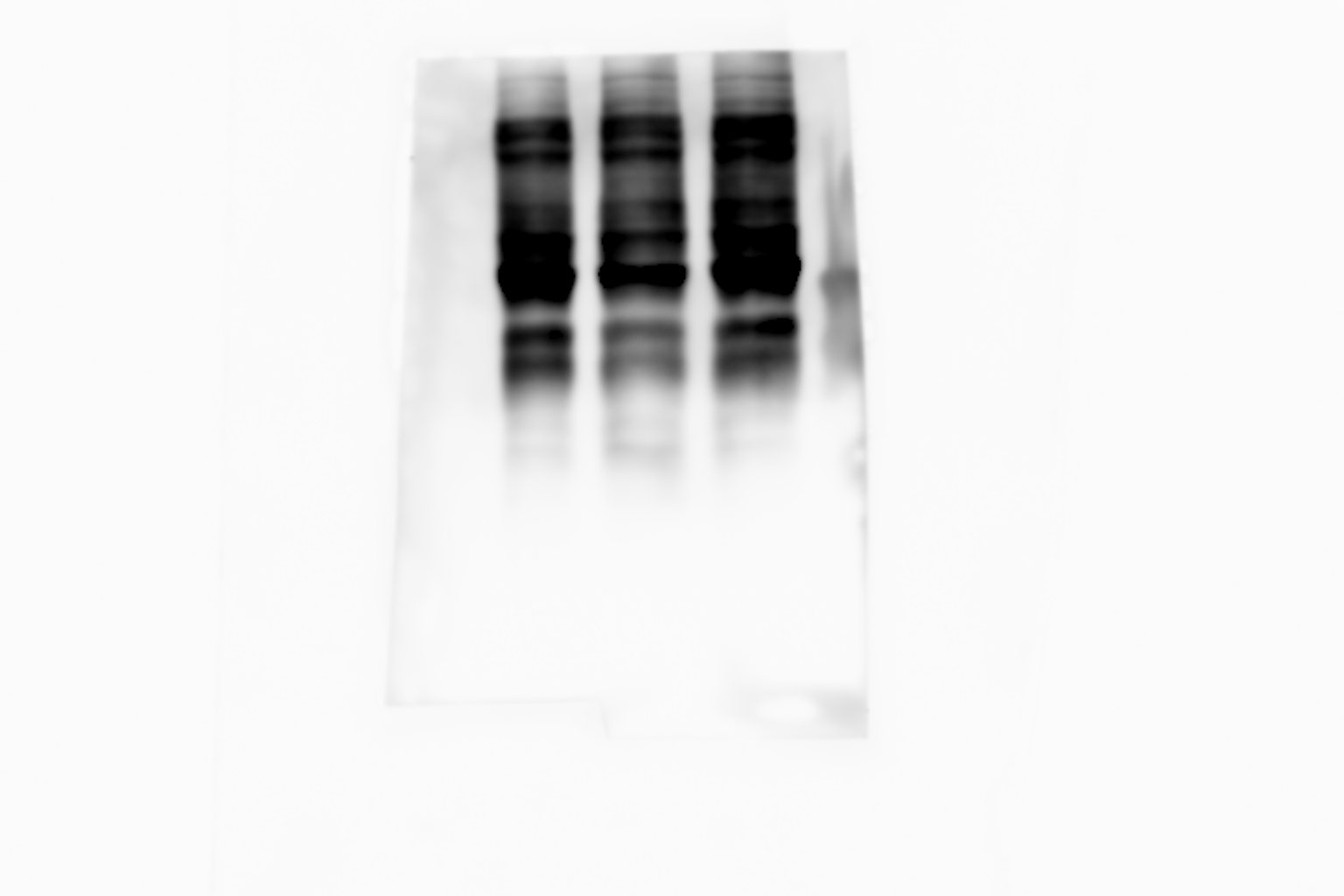

Supplement: Supplementary file 1 [file biology-15-00594-s001.zip › biology-4201171-supplementary/Original images for W.B_Biology/Fig.4A_C2C12/Fig.4A_C2C12_STAT5b_v2.tif]

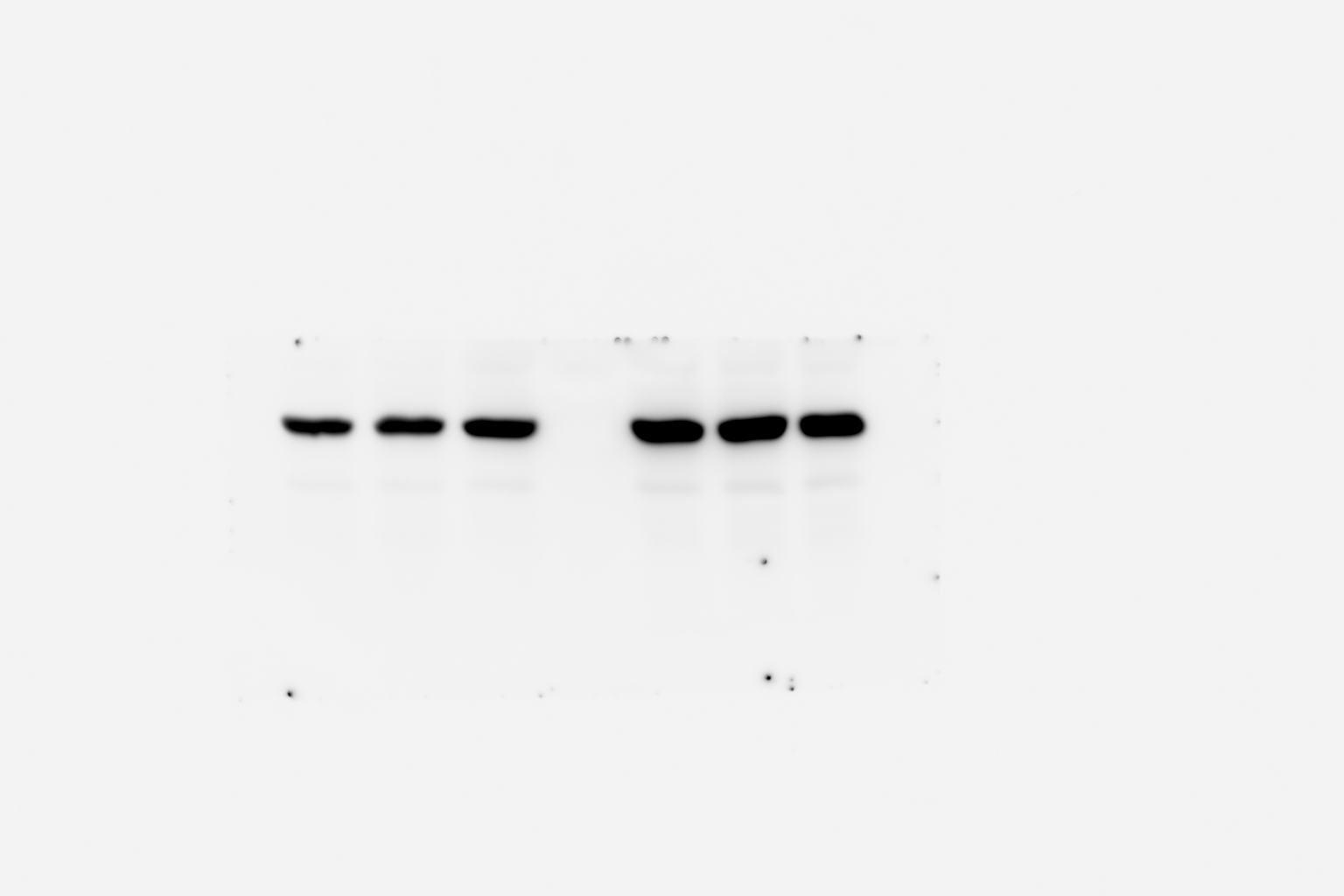

Supplement: Supplementary file 1 [file biology-15-00594-s001.zip › biology-4201171-supplementary/Original images for W.B_Biology/Fig.4A_C3H10/Fig.4A_C3H10_beta-actin-1_v2.tif]

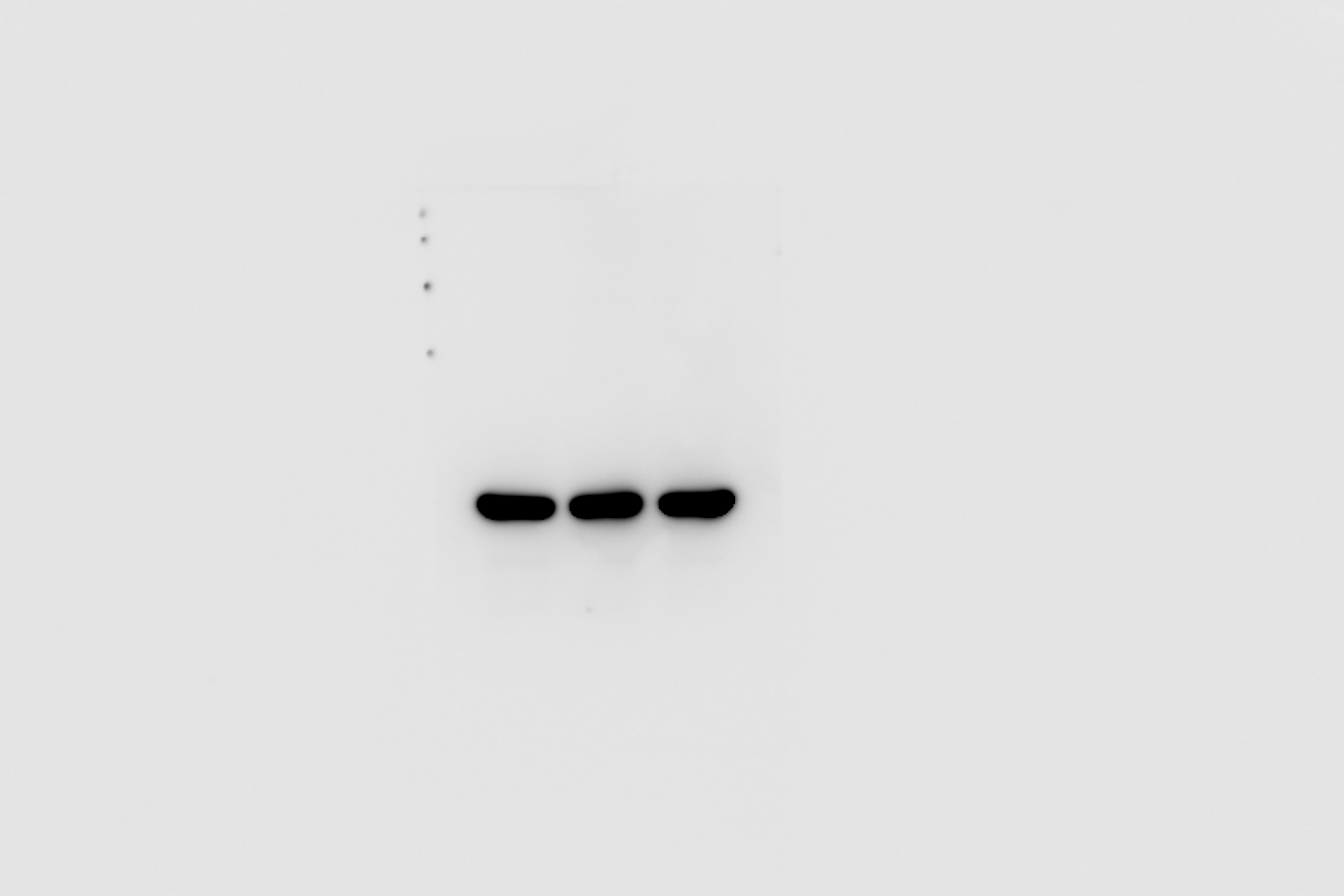

Supplement: Supplementary file 1 [file biology-15-00594-s001.zip › biology-4201171-supplementary/Original images for W.B_Biology/Fig.4A_C3H10/Fig.4A_C3H10_beta-actin-2_v2.tif]

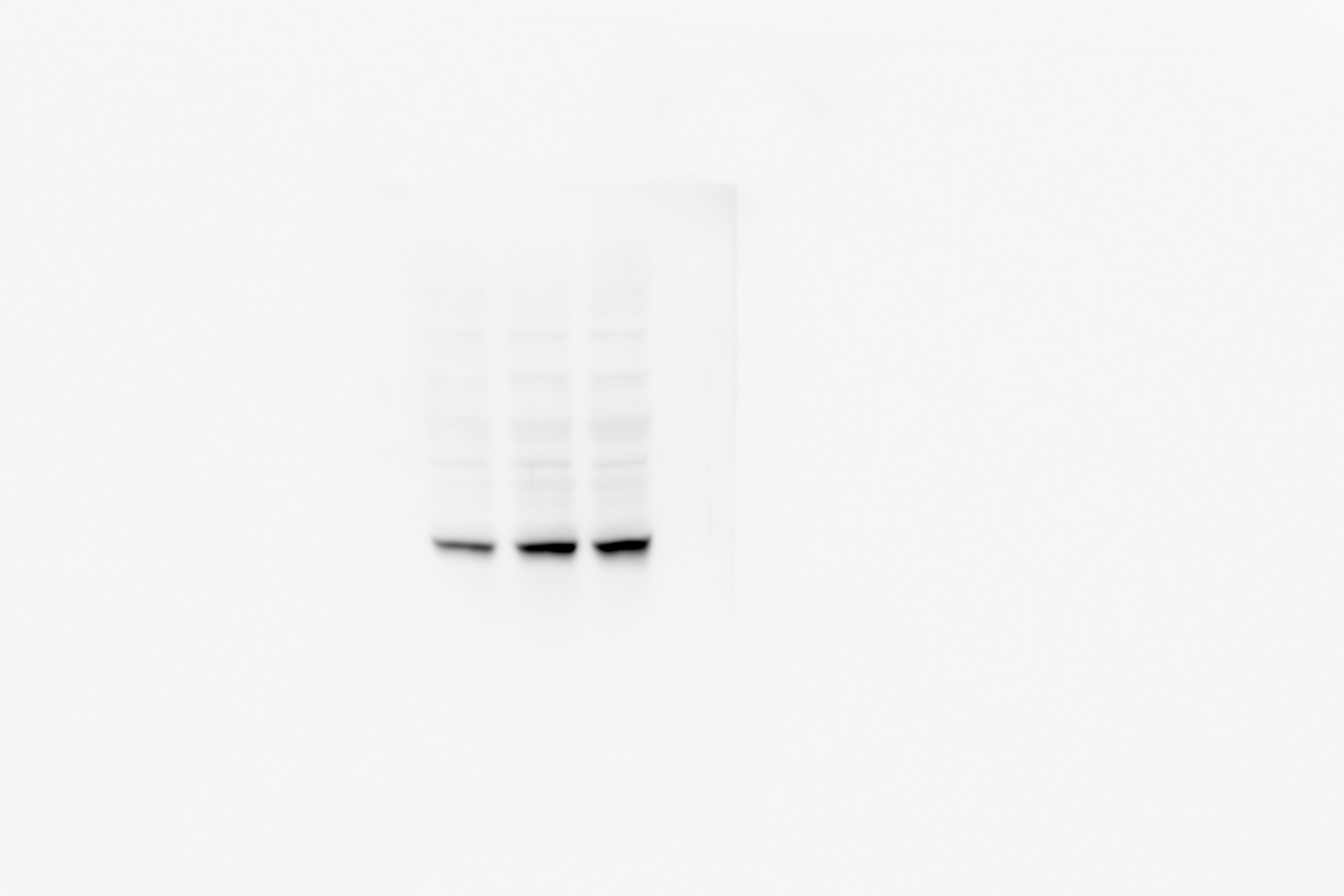

Supplement: Supplementary file 1 [file biology-15-00594-s001.zip › biology-4201171-supplementary/Original images for W.B_Biology/Fig.4A_C3H10/Fig.4A_C3H10_BMP7_v2.tif]

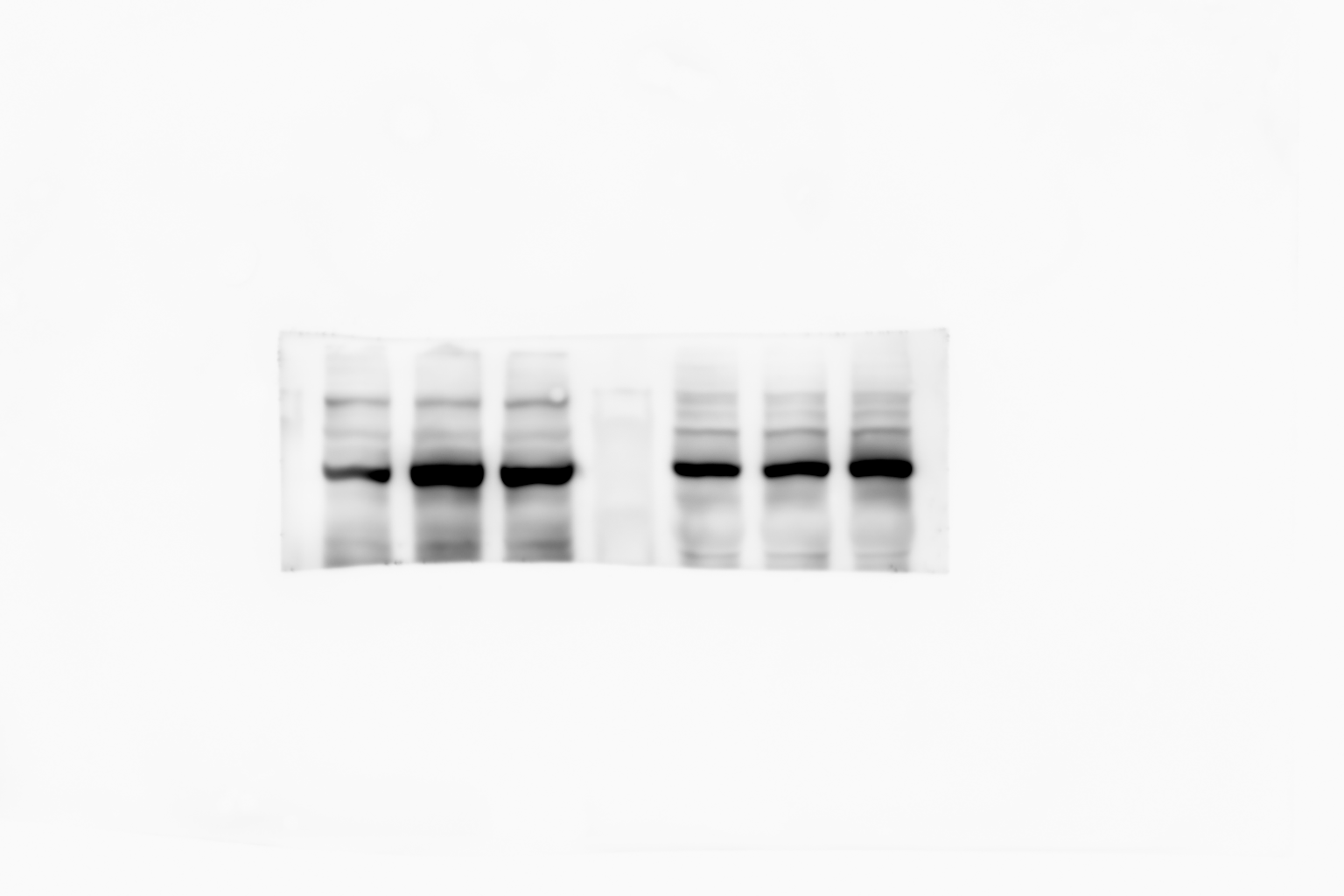

Supplement: Supplementary file 1 [file biology-15-00594-s001.zip › biology-4201171-supplementary/Original images for W.B_Biology/Fig.4A_C3H10/Fig.4A_C3H10_GHR_v2.tif]

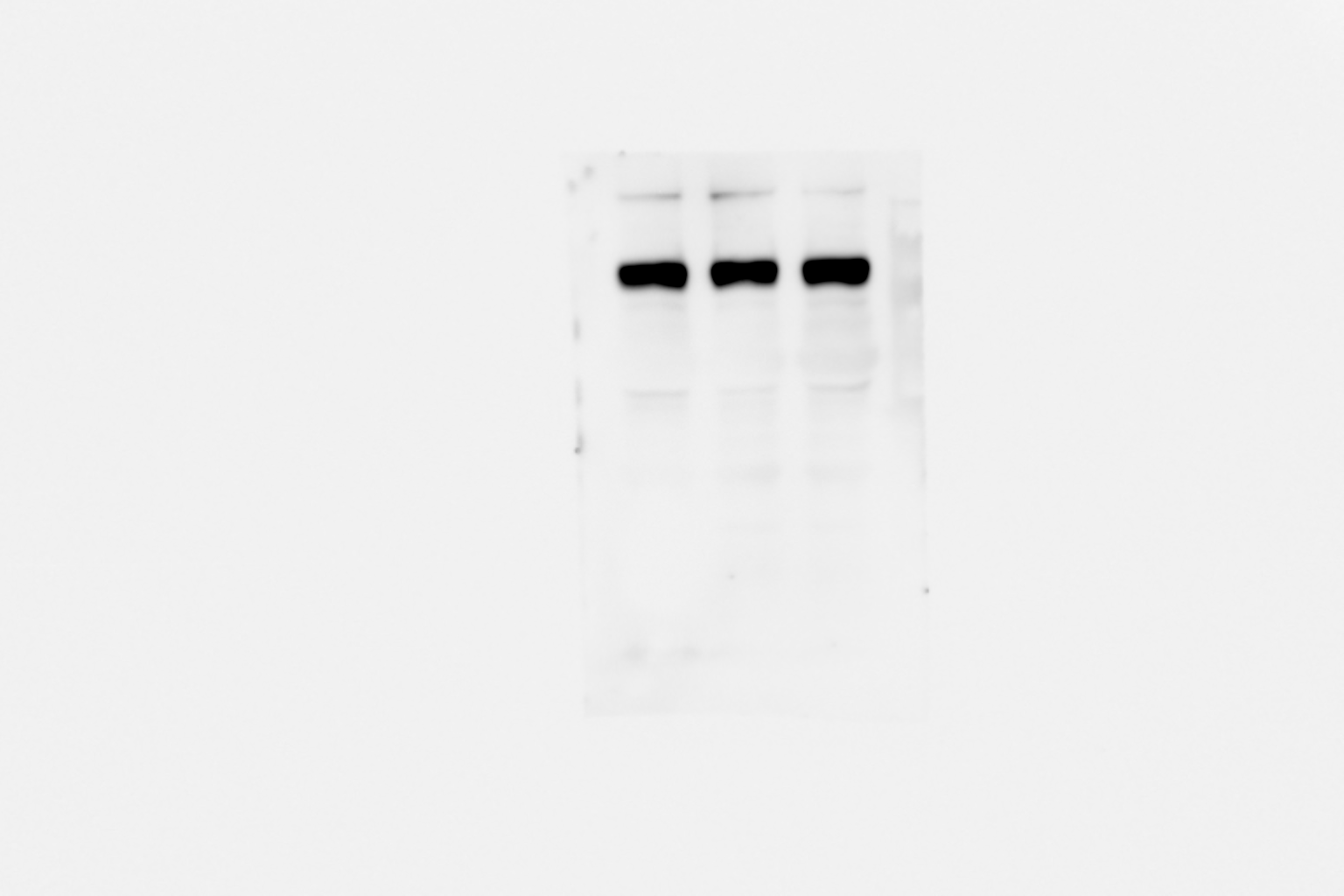

Supplement: Supplementary file 1 [file biology-15-00594-s001.zip › biology-4201171-supplementary/Original images for W.B_Biology/Fig.4A_C3H10/Fig.4A_C3H10_IGF-1Rbeta_v2.tif]

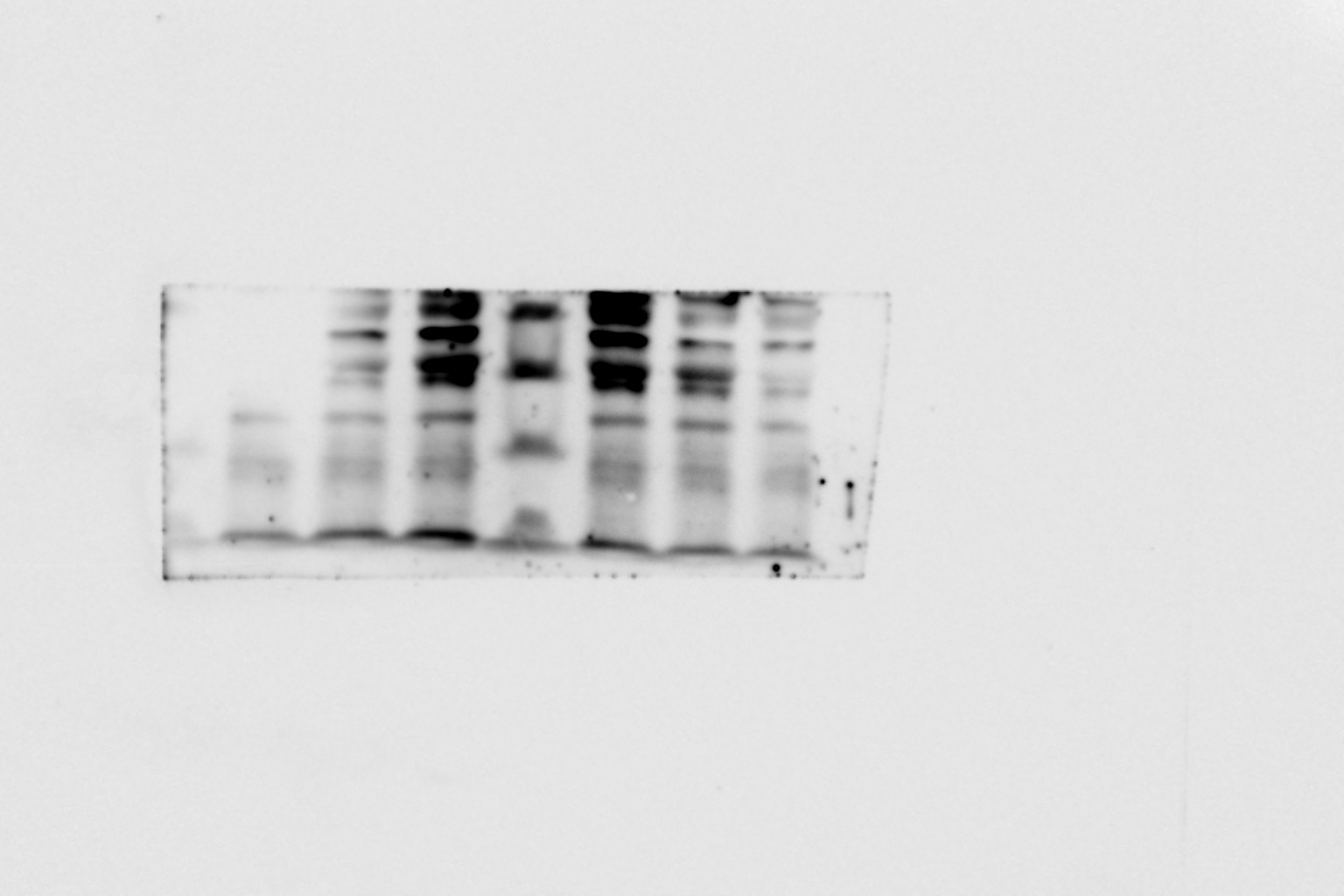

Supplement: Supplementary file 1 [file biology-15-00594-s001.zip › biology-4201171-supplementary/Original images for W.B_Biology/Fig.4A_C3H10/Fig.4A_C3H10_IGF-1_v2.tif]

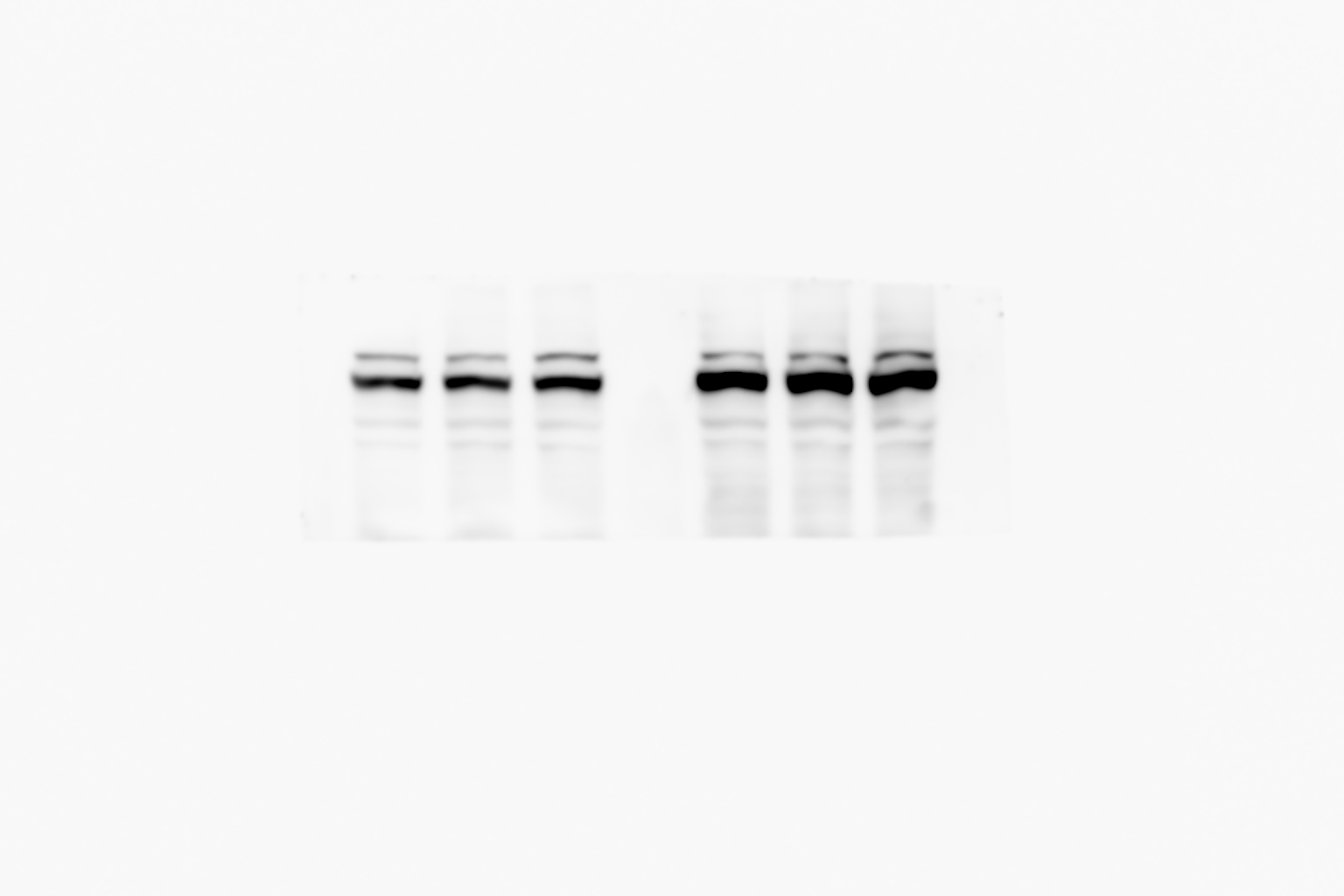

Supplement: Supplementary file 1 [file biology-15-00594-s001.zip › biology-4201171-supplementary/Original images for W.B_Biology/Fig.4A_C3H10/Fig.4A_C3H10_JAK2_v2.tif]

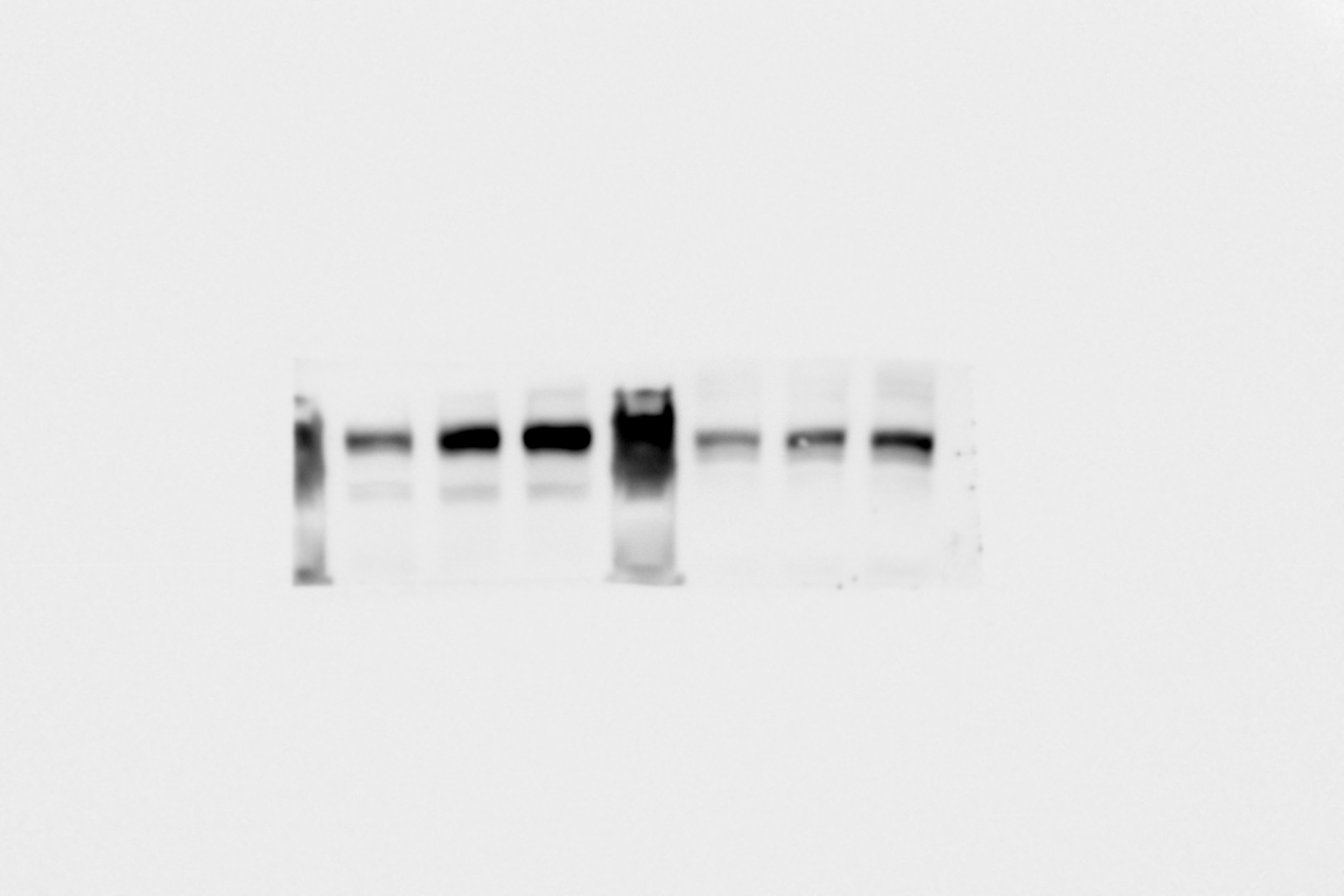

Supplement: Supplementary file 1 [file biology-15-00594-s001.zip › biology-4201171-supplementary/Original images for W.B_Biology/Fig.4A_C3H10/Fig.4A_C3H10_pIGF-1Rbeta_v2.tif]

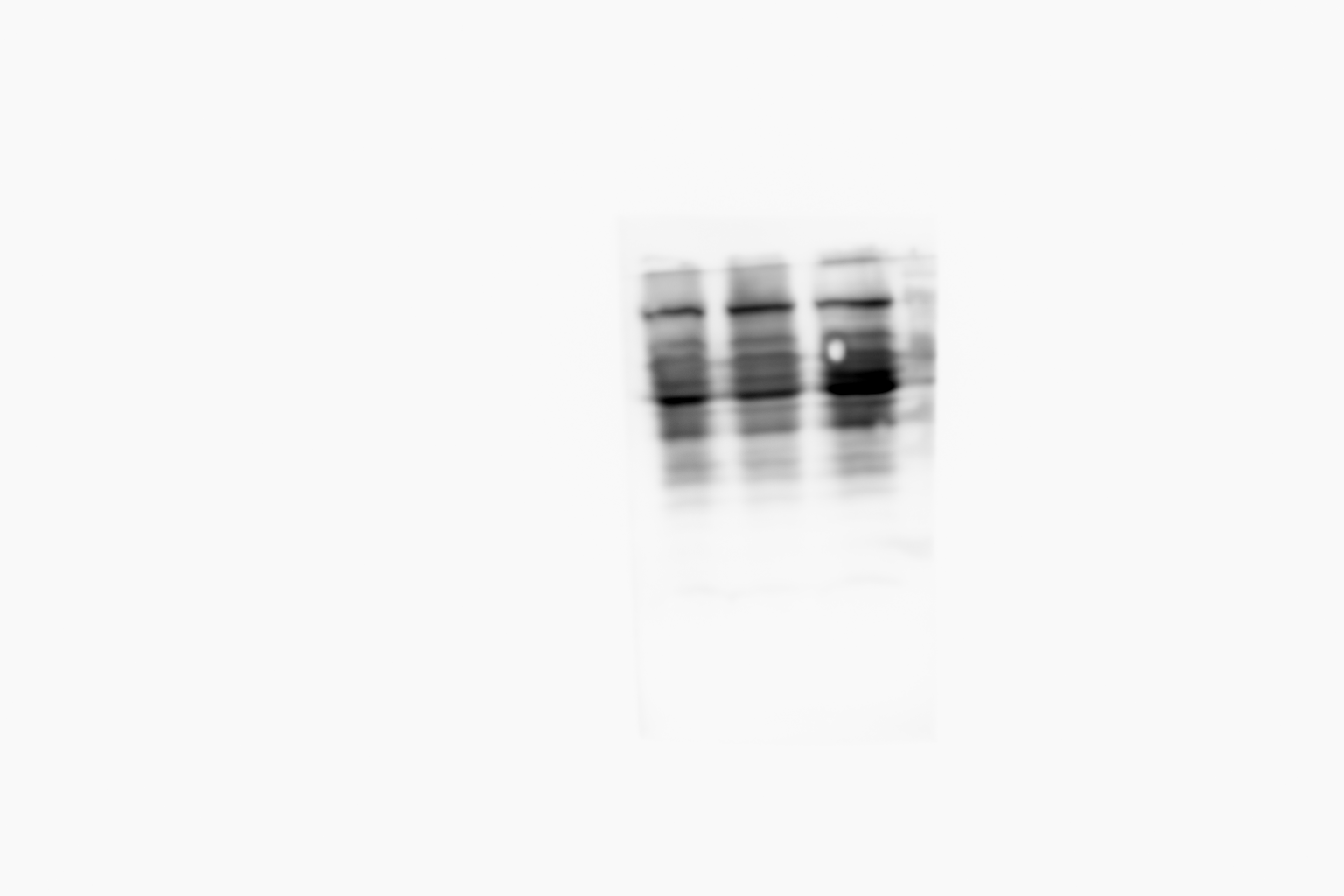

Supplement: Supplementary file 1 [file biology-15-00594-s001.zip › biology-4201171-supplementary/Original images for W.B_Biology/Fig.4A_C3H10/Fig.4A_C3H10_pJAK2_v2.tif]

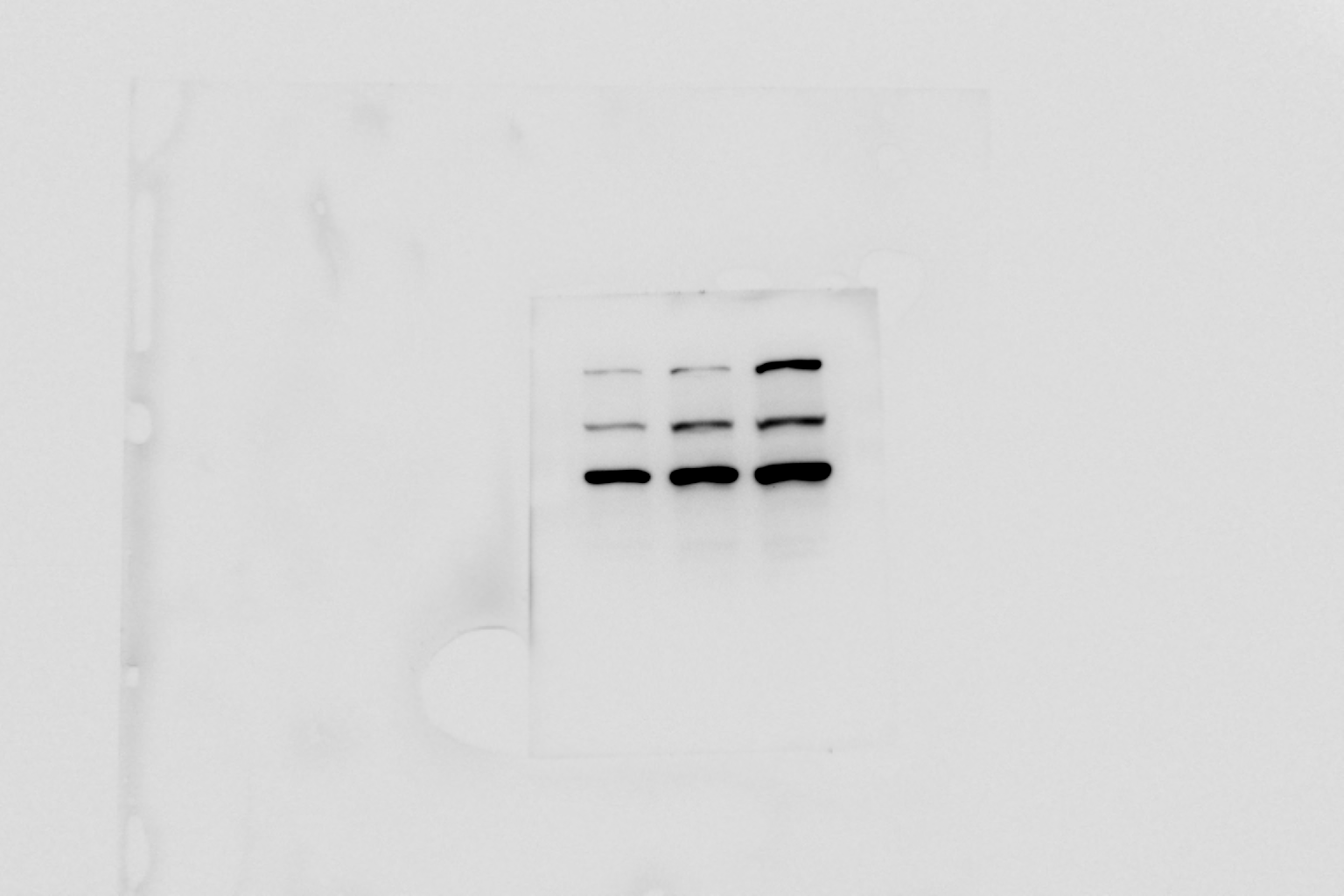

Supplement: Supplementary file 1 [file biology-15-00594-s001.zip › biology-4201171-supplementary/Original images for W.B_Biology/Fig.4A_C3H10/Fig.4A_C3H10_pSTAT5_v2.tif]

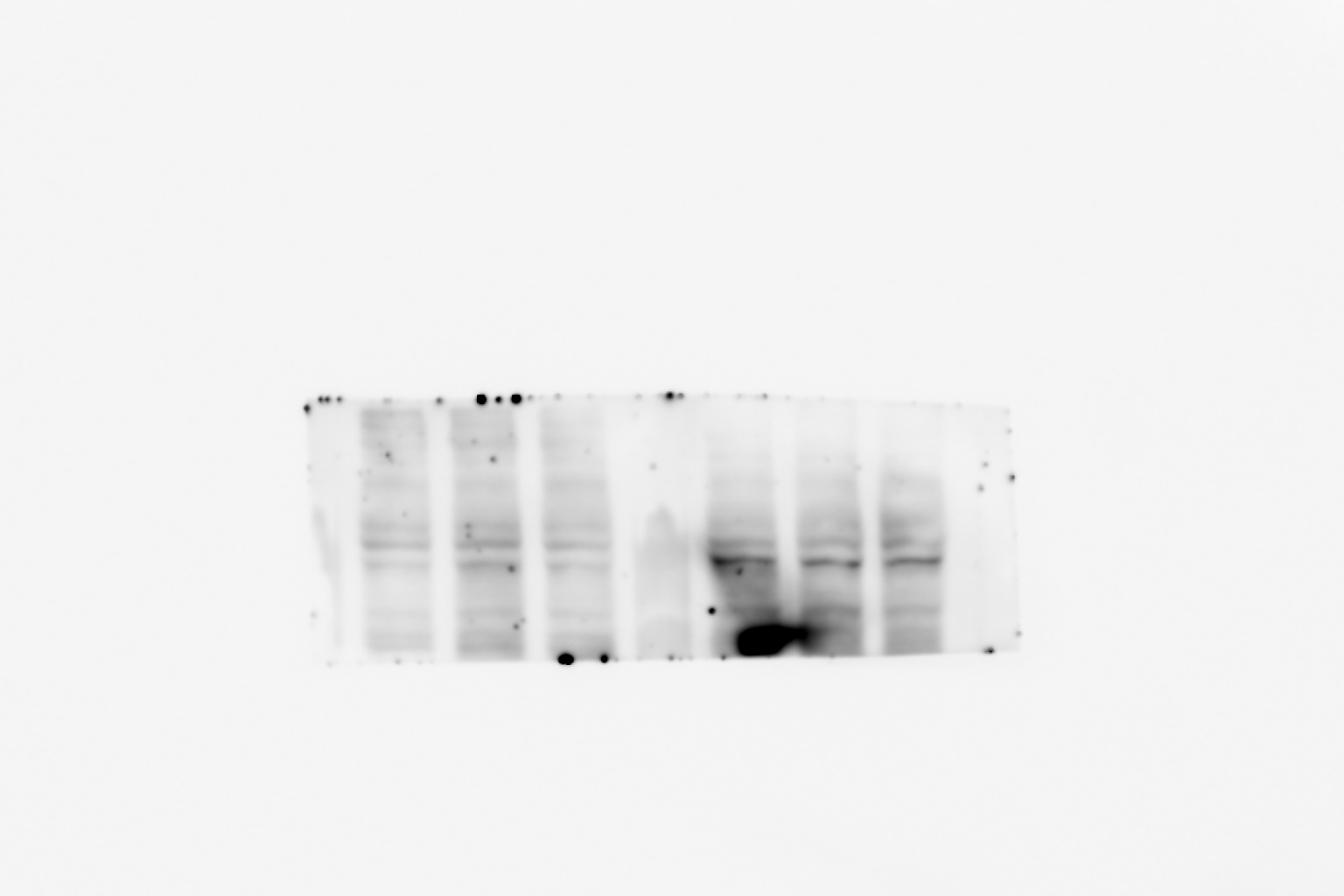

Supplement: Supplementary file 1 [file biology-15-00594-s001.zip › biology-4201171-supplementary/Original images for W.B_Biology/Fig.4A_C3H10/Fig.4A_C3H10_STAT5b_v2.tif]
